# Evaluating the Origins of Aerobic Oxidation Catalysis with TAM-3, a MOF with Accessible Co(II) Sites and Large Pores

**DOI:** 10.1021/acscatal.5c01083

**Published:** 2025-06-02

**Authors:** Aishanee Sur, Subham Sarkar, Nicholas B. Jernigan, Nattamai Bhuvanesh, David C. Powers

**Affiliations:** Department of Chemistry, 2655 Texas A&M University, College Station, Texas 77843, United States

**Keywords:** MOF catalysis, heterogeneous catalysis, C−H functionalization, autoxidation, kinetic isotope effects

## Abstract

Metal-organic frameworks (MOFs) are attractive platforms that merge concepts of homogeneous and heterogeneous catalysis. Catalyst design and optimization are enabled by an array of synthetic methods that offer independent control over the local chemical structure of lattice-embedded metal ions (i.e., ligand identity and geometry) and the long-range materials properties (i.e., porosity). Establishing the origin of catalytic activity in MOF-promoted reactions remains a significant challenge: The relative rates of catalyst turnover and substrate diffusion dictate the extent to which interstitial sites are accessible and operational in catalysis. To minimize the contributions of surface sites in catalysis, materials with large pore dimensions are often sought, however, the impact of pore expansion on the origins of catalytic activity is similarly challenging to establish. Here, we describe TAM-3, a Co­(II) based MOF with accessible metal sites supported by a facially coordinating *tris*-tetrazole ligand set. TAM-3 features large channel-like pores (17 × 23 Å) and promotes aerobic C–H oxidation and olefin epoxidation. Using a set of simple kinetics experiments, based on the analysis of kinetic isotope effects and olefin oxidation diastereoselectivities, we demonstrate that despite the large pores, interstitial metal ions do not significantly contribute to the observed substrate oxidation. This study highlights the importance of conducting kinetic experiments to assess the origin of apparent catalytic activity with MOFs and the challenge of harnessing reactive oxidants with microporous catalyst materials.

## Graphical abstract

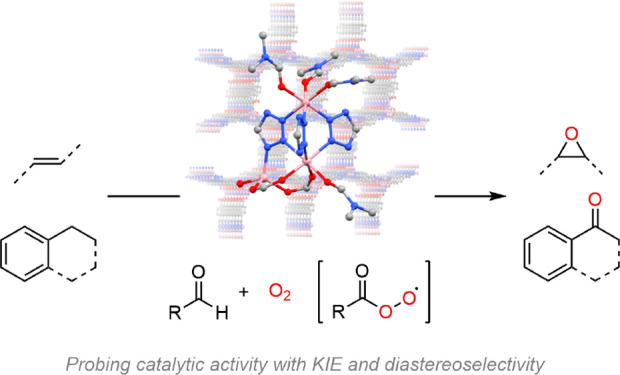

## Introduction

Metal-organic frameworks (MOFs) provide a platform to bridge the conceptual and practical gaps between homogeneous and heterogeneous catalysts. Sophisticated synthetic methods have been developed to incorporate metal ions with specific oxidation states and coordination geometries, which are hallmarks of transition metal catalysis, into MOF lattices.^1–6^ Simultaneously, the material properties and porosity can often be systematically manipulated by judicious linker design,^7^ crystallization protocol,^8^ and postsynthetic processing.^9,10^ As a result, large families of MOFs have been deployed in catalysis, with applications ranging from fine-chemical synthesis, commodity upgrading, to energy conversion chemistry.^11–14^

Application of MOFs in catalysis confronts a complex kinetic landscape.^15–17^ In order to utilize the high density of potential active sites, substrates and reagents must fully diffuse into the interstices of the lattice (i.e., interstitial catalyst sites). If the intrinsic rate of catalyst turnover exceeds that of diffusion, only sites at or near the surface (i.e., interfacial sites) will be involved in catalysis. While there is a rich history of successes in surface organometallic chemistry, efficient access to the interstices of MOFs is needed in order to leverage unique opportunities, such as confinement effects, as a catalyst design element. In addition to surface sites, potential solution-phase reactions (either background reactions or MOF-initiated pathways) may contribute to the overall reaction rate (Figure 1a). In concept, the catalyst turnover frequency (TOF) can be modulated by controlling the coordination environment of lattice-embedded active sites and the rate of diffusion can be controlled by tuning material porosity. In practice, the relative rates of these processes are challenging to experimentally determine, and as a result the origin of catalysis in MOF-promoted reactions is rarely experimentally evaluated.

**Figure 1:**
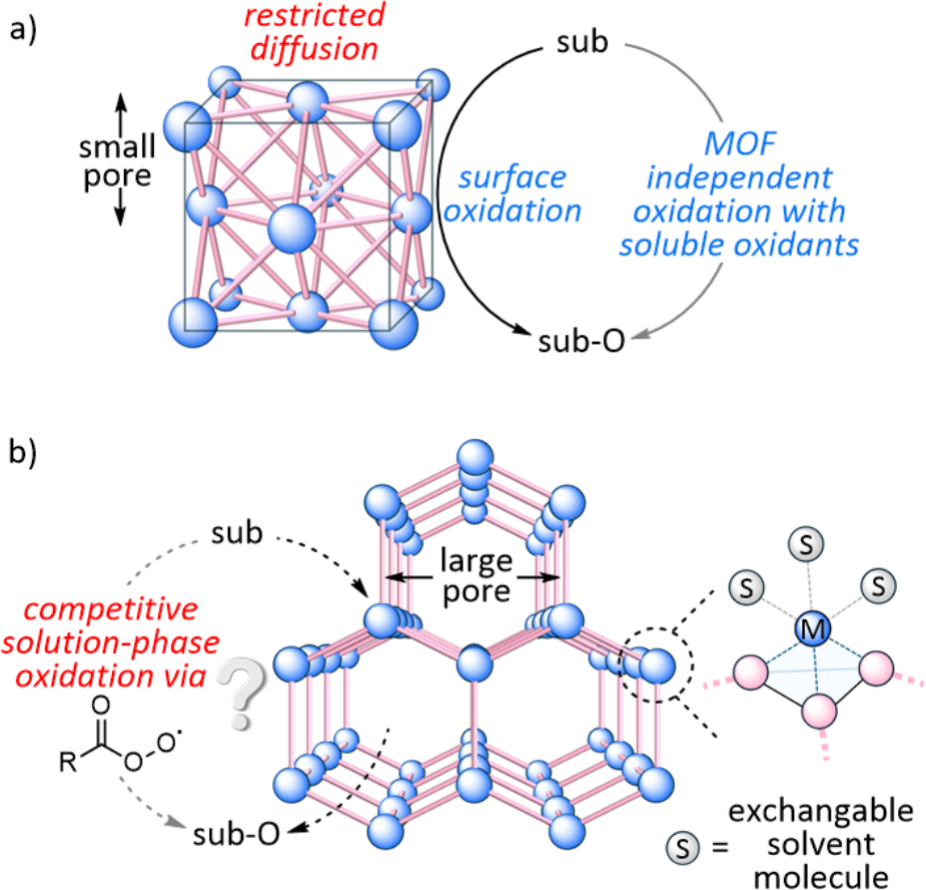
(a) Small pores can suppress MOF-centered catalytic activity when solution phase substrate oxidation is available. (b) Synthetic tuning of the pore structure and the metal coordination center can be utilized to construct a potential MOF catalyst, but kinetic experiments are essential to ascertain the origin of the observed activity.

Single-component diffusivities are routinely measured for gaseous small molecules and substrate vapors using fully activated porous materials.^18–20^ While small guests typically confront low barriers to diffusion in porous solids, condensed-phase substrates confront more significant barriers.^21,22^ For example, while the diffusion coefficient of methane (kinetic diameter = 3.8 Å) in MOF-5 is 1.7 × 10^–7^ m^2^/s, that of benzene (kinetic diameter = 5.8 Å) is 2.0 × 10^–9^ m^2^/s, determined from volumetric gas/vapor adsorption experiments.^21^ When considering catalytic reactions carried out in solution, porous media are completely solvated. Thus, the crystallographically determined pore structure and single-component diffusivities are poor proxies for the effective porosity experienced by diffusing substrates and reagents: Unlike gas phase chemistry, the mean-free-path of a guest in a solvated pore is dictated by solvation more than the underlying pore structure.^23^ In addition to restricted intracrystalline diffusion, the barriers to entry and egress (i.e., intercrystalline diffusion) are often significant as well.^24^ In the context of catalysis, indirect data such as the results of steric sieving experiments, are often used to guide the development of appropriate pore dimensions. To pursue rational catalyst design, and to evaluate the origin of reported catalytic activity, experimental approaches to evaluate the relative contributions of interstitial, interfacial, and solution-phase processes are critical.

Oxygen-atom transfer (OAT) reactions, especially under aerobic oxidation conditions, have been broadly pursued with MOF catalysis.^25–30^ Recently, we advanced kinetic probes based on relative kinetic isotope effects and olefin epoxidation diastereoselectivities to examine the origin of activity in MOF-promoted aerobic oxidations. Specifically, we demonstrate that MIL-125-Cu_2_O_2_, a MOF that had been advanced as a catalyst for aerobic oxidation at interstitial Cu_2_ sites, had no direct role in substrate functionalization but instead served as a radical chain initiator for solution-phase autoxidation chemistry.^31^ Given the fairly small pores of this Cu_2_-based MOF (i.e., ∼6 Å), we viewed the lack of *bona fide* MOF catalysis as potentially due to restricted diffusion. Here, we describe the catalytic activity of a new tripodal Co­(II)-based MOF, TAM-3, featuring large channel pores (17 × 23 Å). The tripodal Co­(II) sites in TAM-3 mimic well-established molecular platforms for OAT catalysis, which are suggested to proceed via reactive Co­(IV)-oxo or Co­(III)-peroxy intermediates and the presence of multiple solvent-derived ligands was viewed as useful to accessing unsaturated sites for substrate activation (Figure 1b).^32–35^ TAM-3 is efficient in promoting OAT chemistry under aerobic conditions. Despite the large pores and accessible metal sites, results of both kinetic isotope effect analysis and olefin oxidation diastereoselectivity experiments indicate that the MOF does not significantly contribute to the observed oxidation chemistry. These results highlight the challenges that must be overcome to develop *bona fide* MOF catalysts for condensed-phase reactions and serve as a call for new models to understand the impact of lattice solvation of the kinetics landscape relevant to MOF catalysis.

## Results and Discussion

During our investigation of MOF-catalyzed C–H functionalization, we were intrigued by the opportunity to translate the chemistry of molecular pseudotetrahedral metal oxo and oxyl complexes^36–41^ and the efficiency of Co­(II) catalysts in oxygen-atom transfer (OAT) chemistry^42–47^ into MOF-based catalysts. Based on the effectiveness of azolate-based linkers in generating MOFs with pseudotetrahedral metal sites,^48,49^ we synthesized TAM-3, a Co­(II)-based MOF with 4-tetrazolebenzoic acid (H_2_TBA). Heating a DMF/H_2_O solution of CoCl_2_·6H_2_O, H_2_TBA, and ammonium formate at 120 °C for 3 days resulted in the formation of purple single crystals of [Co_3_(TBA)_3_(DMF)_4_], which we refer to as TAM-3 (Texas A&M-3) (Figure 2a). SEM of the as-synthesized TAM-3 showed a homogeneous distribution of prismatic single crystals that aggregate to produce larger fan-shaped structures, throughout the material (Figure S1). Single-crystal X-ray diffraction showed that TAM-3 crystallizes in the P2_1_/n space group with an asymmetric unit comprised of three Co­(II) ions, three TBA linkers, and four coordinated DMF ligands (Figure 2b). The extended framework displays sqp topology and the porosity (vide infra) arises from the presence of extended hexagonal channels (17 × 23 Å; Figure 2c). Each of the three Co­(II) centers in the asymmetric unit resides in a unique coordination environment. Co1 and Co2 centers display pseudooctahedral coordination while Co3 displays pseudotetrahedral coordination: Co1 is coordinated by three facially disposed tetrazole donors and three DMF ligands; Co2 is coordinated by three facially disposed tetrazole donors, two carboxylate donors, and one DMF ligand; and Co3 is coordinated by three carboxylate donors and one tetrazole donor. The TBA linkers display multiple coordination modes: Two of the TBA linkers engage in μ_2_–η^1^:η^1^ and μ_2_–η^1^:η^1^:η^0^ coordination of the carboxylate and tetrazole donors, respectively, while one of the TBA linkers engages in a nonbridging η^1^:η^0^ coordination of the carboxylate donor and μ_3_–η^1^:η^1^:η^1^ coordination of the tetrazole donor. A similar framework, with slightly different local Co coordination, was recently reported by Co­(II) metathesis into a Mn­(II) material.^50^

**Figure 2:**
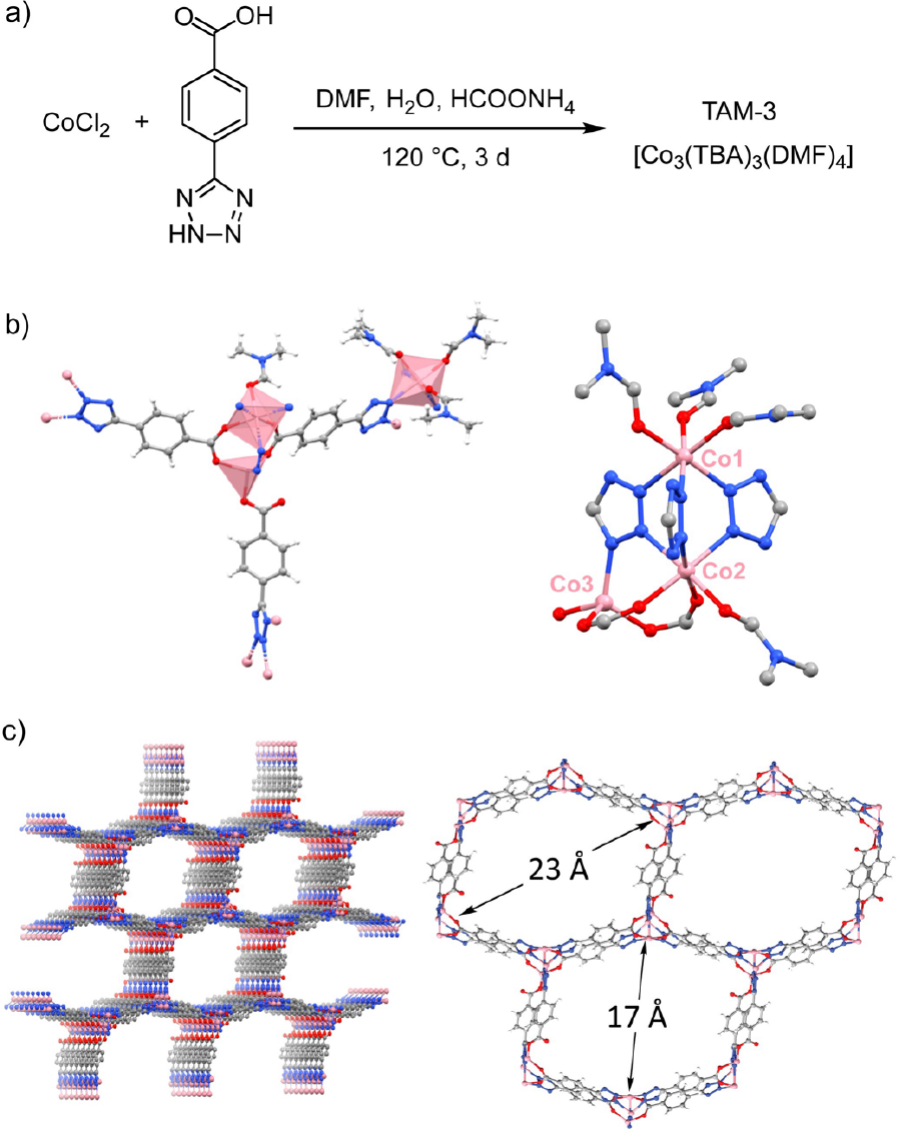
(a) Solvothermal combination of Co­(II) with H_2_TBA in the presence of ammonium formate affords TAM-3. (b) Asymmetric unit features three unique Co­(II) sites, two of which are octahedrally coordinated and one of which is tetrahedrally coordinated. (c) TAM-3 features pseudohexagonal 1D channel pores with 17 Å and 23 Å diagonals (DMF molecules omitted for clarity).

Infrared (IR) spectroscopy (Figure 3a) and thermal gravimetric analysis (TGA, Figures 3b and S2) indicate the presence and lability of coordinated DMF ligands, respectively. PXRD analysis of as-synthesized TAM-3 is consistent with the diffraction pattern calculated from the single-crystal parameters (Figure 3c). The peak intensities observed for TAM-3 reflect strong preferred orientation because the material cannot be finely ground without loss of crystallinity. The peak positions and intensities were also found to depend on lattice solvation. TAM-3 loses crystallinity upon desolvation from coordinating solvents like acetonitrile but retains crystallinity when dried from dichloroethane (DCE) via slow evaporation. Further activation of the DCE-derived samples under active vacuum also led to loss of crystallinity, which prevented gas sorption analysis.

**Figure 3:**
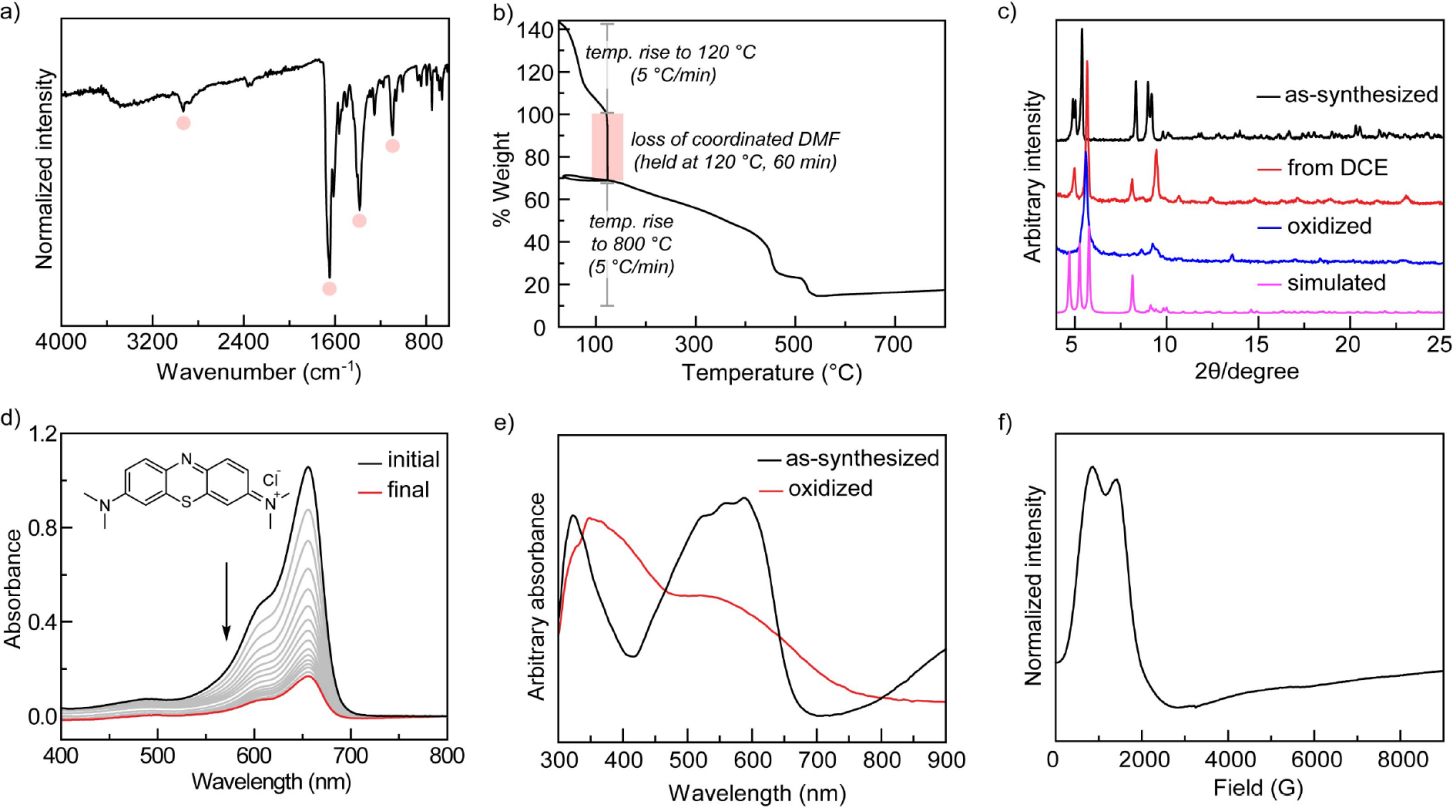
(a) IR spectrum of as-synthesized TAM-3 showing stretches for bound DMF molecules. (b) Thermogravimetric analysis of as-synthesized TAM-3 showing 30% weight loss corresponding to the bound DMF molecules at 120 °C. (c) PXRD patterns for TAM-3 (**blackline**) as-synthesized, (**red line**) DCE exchanged and dried, (**blue line**) mCBPA treated and dried, (**pink line**) simulated from single-crystal diffraction data. (d) Methylene blue uptake by TAM-3 tracked by UV–vis spectroscopy. (e) Diffuse reflectance UV–vis spectra of (**black line**) as-synthesized, and (**red line**) mCPBA treated TAM-3. (f) EPR spectrum of as-synthesized TAM-3 at 5 K.

To confirm the presence of large, substrate accessible pores, dye-uptake experiments were carried out.^51^ Air-dried TAM-3 crystals were added to acetone solutions of methylene blue (MB) or crystal violet (CV) and the adsorption of these dyes was assessed by UV-vis spectroscopy of the supernatant. While CV (molecular volume ∼ 380 Å^3^) was adsorbed only slightly (Figure S3), MB (molecular volume ∼ 270 Å^3^) was readily adsorbed by TAM-3 (59 mmol/mol) (Figure 3d), showing that it has sufficient porosity for the diffusion of small organic molecules.^52^ In addition, 4-aminobenzonitrile was found to diffuse into TAM-3 and could be reversibly expelled upon re-exposure to acetonitrile (Figure S4).

The diffuse reflectance UV-vis spectrum of as-synthesized TAM-3 is pictured in Figure 3e. The intense absorbance centered at 320 nm is due to the TBA ligand. A lower energy absorption envelop, with maxima at 524, 558, and 588 nm, is characteristic of the d-d transitions expected for a tetrahedrally coordinated Co­(II) ion (Figure 3e).^53^ Lower-intensity features ∼500 nm for octahedral Co­(II) centers are likely to be buried under the strong absorption of tetrahedral sites. The EPR spectrum of TAM-3 recorded at 5 K displays two overlapping signals around 1000 G, which are consistent with the presence of both high-spin octahedral and tetrahedral Co­(II) centers (Figure 3f).^54,55^

Treatment of TAM-3 with mCPBA resulted in an immediate and irreversible color change from purple to brown. Diffuse reflectance UV-vis spectroscopy of the MOF following treatment with mCPBA displayed a decrease in the overall absorption intensity and loss of the spectral features around 550 nm, which we had attributed to the octahedral and tetrahedral Co­(II) centers, respectively (Figure 3e). The PXRD pattern following mCPBA treatment demonstrated that crystallinity was not lost upon oxidation (Figure 3c). Oxidation of TAM-3 is accompanied by complete disappearance of the EPR signals (5 K), which is consistent with conversion of high-spin Co­(II) sites to low-spin octahedral Co­(III). Together, these data indicate that the Co­(II) nodes of TAM-3 are accessible to soluble oxidants and that all the Co­(II) is oxidized, not just surface sites.

The combination of large channel-shaped pores, demonstrated redox chemistry of lattice ions, and presence of pseudotetrahedral Co­(II) centers that resemble solution-phase OAT catalysts led us to explore application of TAM-3 in OAT catalysis. We were particularly interested in the potential for TAM-3 to accomplish OAT catalysis under aerobic conditions based on our long-standing interest in O_2_ utilization^56–58^ and the frequency with which Co-catalyzed aerobic oxidation is pursued.^59–63^ We have pursued interrupted aldehyde autoxidation chemistry as a strategy for O_2_ utilization in the context of aerobic hypervalent iodine catalysis and Lin^25,26^ and others^64–66^ have utilized these conditions in the context of MOF-promoted oxidation chemistry. In this paradigm, O_2_ engages in radical chain autoxidation via the intermediacy of peracid, peroxy radical, and carboxy radical intermediates.^56^

Oxidation of a small family of olefins under the dual action of O_2_ and isobutyraldehyde in the presence of 2 mol % TAM-3 at 23 °C afforded the corresponding epoxides in high yields (**3a**–**3c**, Figure 4a). Small amounts of ketones, obtained via allylic oxidation, were also observed. Benzylic C–H bonds also underwent oxidation under similar conditions, yielding benzylic ketones along with trace amounts of the corresponding alcohols (**4a**–**4c**, Figure 4a). This selectivity suggests oxidation of the initially formed alcohol to afford the corresponding ketone is facile.^67^ Oxidation of a sp^3^ C–H bond was also obtained for adamantane, yielding 1-adamantanol (**4d**). All reaction components—**1** or **2**, TAM-3, isobutyraldehyde, and O_2_—are required to effect substrate oxidation. However, substrate oxidation activity can be recovered by adding CoCl_2_ instead of TAM-3. The MOF can be recycled: we carried out three catalytic runs for the oxidation of **1a** to **3a** by recycling the same MOF and obtained yields of 87, 78, and 90%, respectively. Diffuse reflectance UV-vis (Figure S5) and EPR (5 K) spectra obtained following aerobic oxidation are similar to those obtained after treatment of TAM-3 with mCPBA.

**Figure 4:**
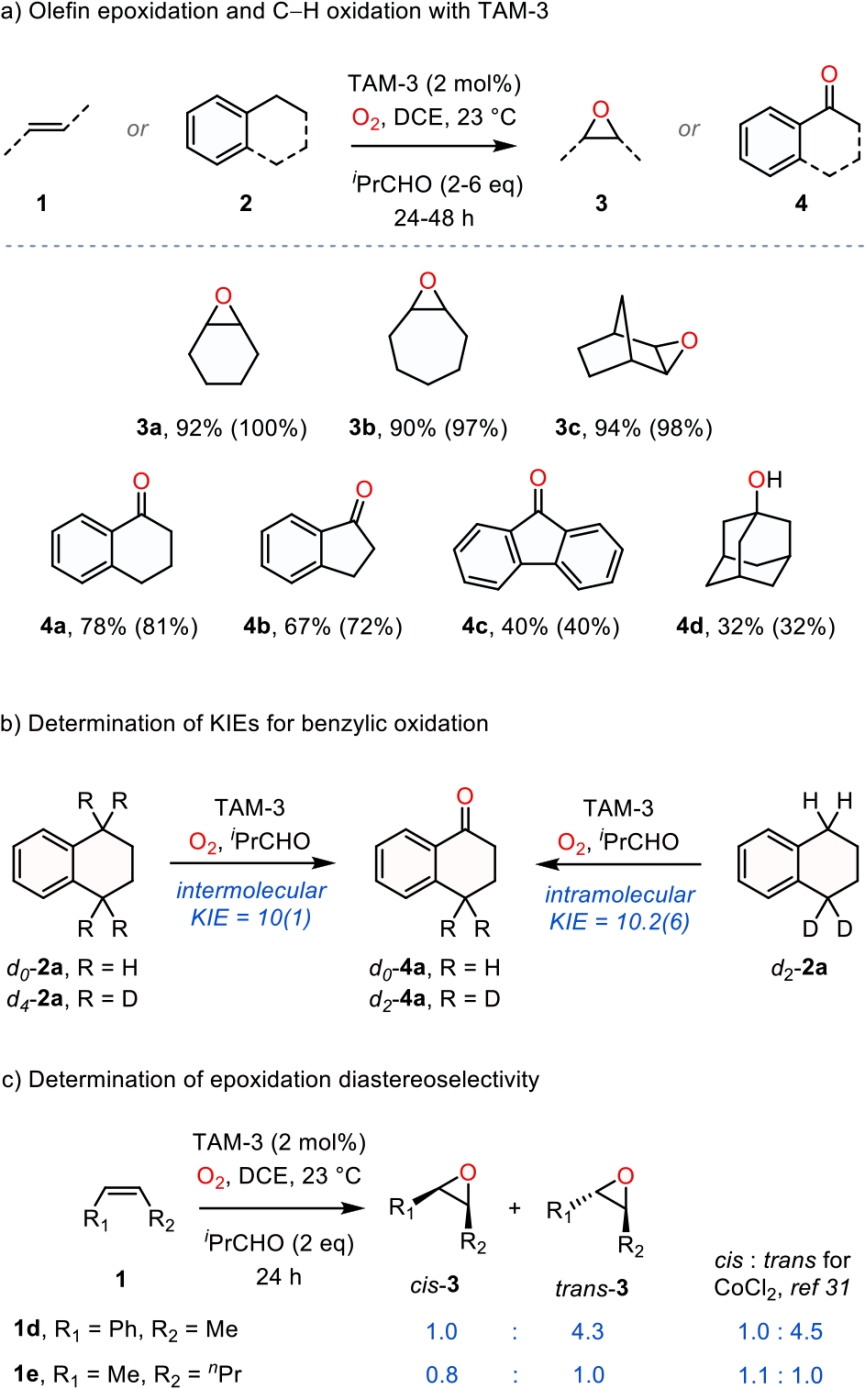
(a) Olefin epoxidation and C–H oxidation in the presence of TAM-3, O_2_, and isobutyraldehyde. (b) Inter- and intramolecular KIE analysis for the aerobic oxidation of benzylic C–H bonds. (c) Diastereoselectivity analysis of aerobic oxidation of *cis*-olefins promoted by TAM-3.

The observation that soluble oxidants can diffuse throughout the network of TAM-3 does not imply that interstitial sites are directly involved in substrate oxidation promoted by TAM-3. We have previously developed tools based on kinetic isotope effects (KIEs) to evaluate the role of MOFs in C–H oxidation reactions.^31^ To apply these experiments to the aerobic oxidation chemistry promoted by TAM-3, we measured the intermolecular KIE for the aerobic oxidation of **2a** to **4a** using a mixture of *d*_0_-**2a** and *d*_4_-**2a** (Figure 4b). The observed KIE for aerobic oxidation in the presence of TAM-3 was found to be 10(1), which is within the uncertainty of the same measurement carried out in the presence of CoCl_2_ (9.9(7)) and CuCl_2_ (10.0(6)). The intramolecular KIE for the oxidation of **2a** to **4a**, measured using *d*_2_-**2a**, was observed to be 10.2(6); similar values were obtained for reactions in the presence of CoCl_2_ (10(1)) and CuCl_2_ (10.2(4)). The identical KIEs, regardless of the initiator used, indicate that a TAM-3-supported oxidant is not involved in the turnover-limiting step of catalysis. These KIEs are consistent with C–H oxidation by soluble oxidants generated as part of a Co­(II)-initiated aldehyde autoxidation manifold. The lack of MOF-based catalysis is striking given the large pores in TAM-3.

In addition to benzylic C–H oxidation, TAM-3 also promotes epoxidation of olefins under aerobic oxidation conditions. Similar to the magnitude of KIEs, olefin oxidation diastereoselectivities provide a sensitive kinetic probe for the identity of the oxidant that engages directly in substrate functionalization.^31^ Importantly, oxidation of *cis*-olefins represents a much more sensitive kinetic probe than *trans*-olefins: High diastereoselectivity is likely to result from functionalization of *trans*-olefins regardless of mechanism due to underlying thermodynamic considerations; erosion of diastereopurity of *cis*-olefins provides a sensitive probe of the synchronicity of bond-forming and -breaking processes. Epoxidation of *cis*-β-methylstyrene (**1d**) in the presence of TAM-3 yielded a 1.0:4.3 mixture of *cis* **-** and *trans*-**3d**. Epoxidation of *cis*-2-hexene (**1e**) in the presence of TAM-3 resulted in a 0.8:1.0 ratio of *cis*-**3e** to *trans*-**3e** (Figure 4c). Similar to the results of KIE studies above, the similarity of the observed diastereoselectivities measured for epoxidation in the presence of TAM-3 to those measured for aerobic epoxidation in the presence of CoCl_2_ (1.0:4.5 for *cis*-**1d** and 1.1:1.0 for *cis*-**1e**) suggests that the Co sites of TAM-3 do not directly participate in olefin epoxidation. In addition to these diastereoselectivities, a plot of [**3a**] vs time displays a significant induction period (i.e., sigmoidal kinetics), further suggesting that the MOF is acting as an autoxidation initiator (Figure S6).

The experiments described here comprise a general strategy to evaluate potential MOF-based catalysts for oxygen-atom transfer (OAT) chemistry (Figure 5). First, one should evaluate whether efficient background reactions (i.e., in the absence of the new porous material or with simple metal salts) are observed. If not, the material plays an explicit role in promoting the observed oxidation reaction. If yes, kinetics experiments should be pursued. Analysis of kinetic isotope effects (KIEs) and olefin oxidation diastereoselectivities (in particular those obtained from oxidation of *cis*-disubstituted olefins) represent sensitive kinetics probes of the mechanism of OAT to substrate. If the results of these kinetics experiments for MOF-promoted reactions differ from those of background reactions, the MOF is an active participant in the catalytic OAT event. If the results of these kinetics experiments for MOF-promoted reactions are the same as those obtained for background reactions, the MOF does not serve as a OAT catalyst, but instead has a different role, such as an initiator for solution-phase oxidation.

**Figure 5:**
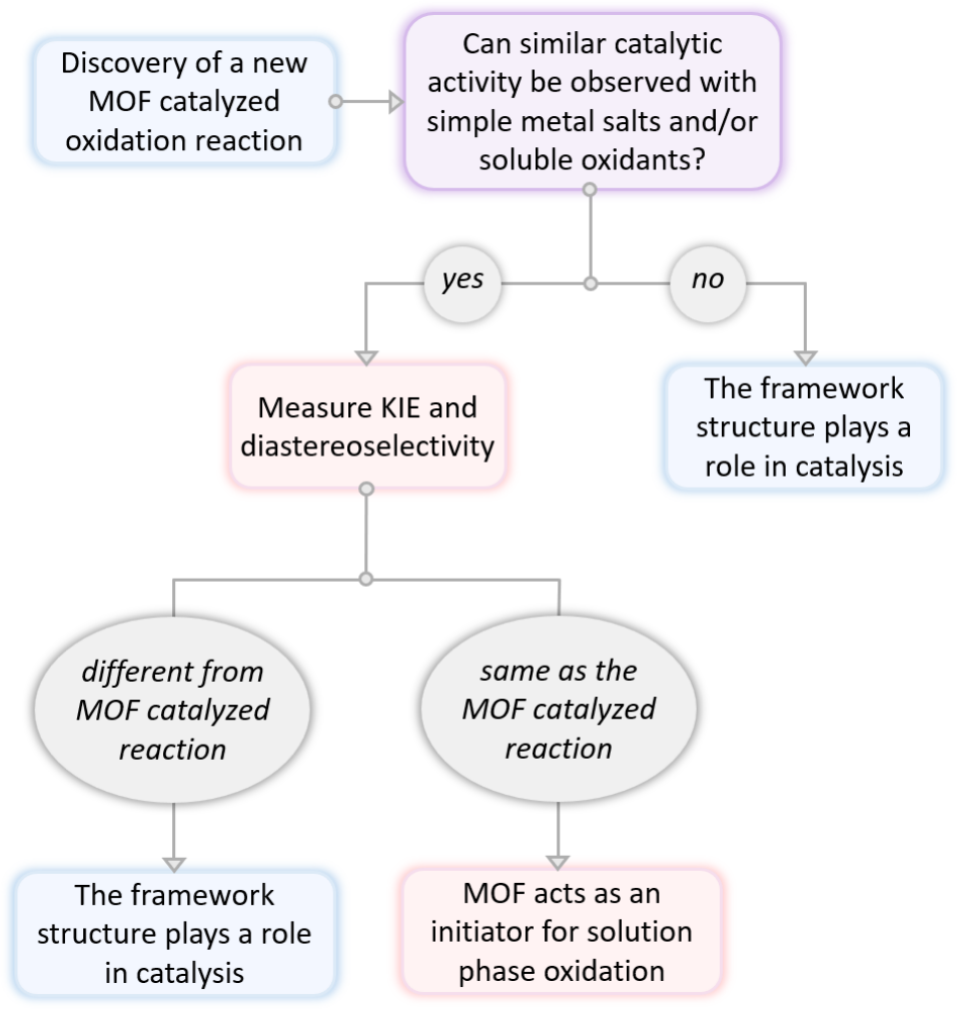
Experimental workflow for evaluating new materials as potential OAT catalysts.

## Concluding Remarks

The local and long-range modularity available in MOF synthesis presents a unique platform to develop heterogeneous catalysts with molecularly defined active sites. The potential to utilize these platforms to achieve challenging catalytic transformations and access unique reaction selectivities has attracted significant attention. Many of these initiatives are predicated on the combination of high-active site density coupled with potential confinement effects to develop active, selective catalyst materials.

Here, we disclose a new MOF, TAM-3, which is comprised of tripodal Co­(II) sites embedded within a honeycomb lattice featuring large columnar pores (17 × 23 Å). We were attracted to this material due to the structural homology of the lattice-confined metal ions and extant solution-phased catalysts. We found that TAM-3 promotes aerobic C–H oxidation and olefin epoxidation chemistry under reaction conditions—i.e., interrupted aldehyde autoxidation—that have become increasingly popular in MOF catalysis. We present two simple kinetics experiments, based on analysis of kinetic isotope effects and olefin oxidation diastereoselectivities, that together indicate that the observed TAM-3 promoted reactions arise from solution-phase autoxidation intermediates and do not involve the intimate participation of lattice-bound ions in the OAT transition states of these reactions. Put differently, the solution-phase oxidation processes, in this case via reactive autoxidation intermediates, are too fast for potential lattice-based mechanisms that require diffusion to compete.^68^

Together, the described data highlight that the presence of large pores is insufficient to ensure interstitial catalysis. In addition, MOFs can undergo structural changes and/or lose crystallinity during the course of a catalytic reaction. As such, kinetic probes are critical to evaluating the origin of apparent catalytic activity. In particular, the role of pore structure in catalysis is important to evaluate when high-yielding solution-phase pathways are accessible. The experimental workflow, based on simple kinetic probes that *do not* require determination of absolute reaction rates, provides a rapid and robust test for potential C–H oxidation catalyst materials. We anticipate that further kinetics assessment will both establish the origin of activity in other MOF-promoted reactions as well as define the design criteria for accessing frameworks that engender rapid diffusion so as to enable *bona fide* interstitial MOF catalysis.

## Materials and Methods

All information detailing the materials and methods employed in this study are detailed in the accompanying Supporting Information.

### Synthesis of TAM-3

A 20 mL scintillation vial was charged with CoCl_2_·6H_2_O (50.0 mg, 0.210 mmol, 1.00 equiv), H_2_TBA (40.0 mg, 0.210 mmol, 1.00 equiv), and ammonium formate (8.00 mg, 0.127 mmol, 0.605 equiv). DMF (1.56 mL) and H_2_O (143 μL) were added to the reaction vessel and the resulting mixture was agitated by sonication until a dark blue solution was obtained. The reaction vessel was placed in an oven preheated at 120 °C for 3 d. The reaction vial was removed from the oven and the hot supernatant was immediately decanted. Fresh DMF (2 mL) was added, and the resulting mixture was stored at 23 °C for 16 h before the DMF was decanted. Additional DMF was added and the soaking (16 h)/decanting cycle was repeated three times, at which point the decanted DMF was colorless. Dark purple crystals of formula [Co_3_(TBA)_3_(DMF)_4_] were obtained. DMF was replaced with DCE (2 mL) and the crystals were soaked for 24 h. The solvent was decanted and fresh DCE (2 mL) was added. After 24 h all the solvent was decanted and the MOF was allowed to air-dry to afford TAM-3 as a purple powder (43 mg, 59% yield). The crystals were characterized by PXRD, IR, TGA and DR-UV-vis spectroscopy.

### General Procedure for Olefin Epoxidation

A 20 mL scintillation vial was charged with the appropriate olefin (0.250 mmol, 1.00 equiv) and DCE (2 mL). TAM-3 (5.16 mg, 5.00 μmol, 2.00 mol %) was added and the vial was fitted with a rubber septum and an O_2_-filled balloon. Isobutyraldehyde (45.6 μL, 0.500 mmol, 2.00 equiv) was added, and the mixture was stirred at 23 °C for 24 h. The reaction mixture was filtered through a Celite plug. The organics were washed with 2 mL deionized water twice and dried over Na_2_SO_4_. Mesitylene was added as an internal standard and the yield was calculated from the ^1^H NMR of the crude reaction mixture.

### General Procedure for C–H Oxidation

A 20 mL scintillation vial was charged with the appropriate substrate (0.250 mmol, 1.00 equiv) and DCE (2 mL). TAM-3 (5.16 mg, 5.00 μmol, 2.00 mol %) was added and the vial was fitted with a rubber septum and an O_2_-filled balloon. Isobutyraldehyde (45.6 μL, 0.500 mmol, 2.00 equiv) was added, and the mixture was stirred at 23 °C for 16 h. A second portion of isobutyraldehyde (45.6 μL, 0.500 mmol, 2.00 equiv) was added, and the mixture was stirred at 23 °C for 16 h. The O_2_ balloons were refilled and a third portion of isobutyraldehyde (45.6 μL, 0.500 mmol, 2.00 equiv) was added, and the mixture was stirred at 23 °C for additional 16 h. The reaction mixture was filtered through a Celite plug. The organics were washed with 2 mL deionized water twice and dried over Na_2_SO_4_. Mesitylene was added as an internal standard and the yield was calculated from the ^1^H NMR of the crude reaction mixture.

## Supplementary Material





## Notes

Experimental procedures and spectroscopic data (PDF)
Crystallographic data of TAM-3 (CIF)
