# Supplementary material for: Evaluating the Origins of Aerobic Oxidation Catalysis with TAM-3, a MOF with Accessible Co(II) Sites and Large Pores
Source: ACS Catal. 2025 Jun 2;15(12):10328–35. doi: 10.1021/acscatal.5c01083 (PMC12186251; doi:10.1021/acscatal.5c01083)
Supplement: Supplementary file 1 [file cs5c01083_si_001.pdf]

Supporting Information

# Evaluating the Origins of Aerobic Oxidation Catalysis with TAM-3, a MOF with Accessible Co(II) Sites and Large Pores

Aishanee Sur, Subham Sarkar, Nicholas B. Jernigan, Nattamai Bhuvanesh,

and David C. Powers*

*Department of Chemistry, Texas A&M University*
*College Station, TX 77843, United States*

Email: powers@chem.tamu.edu

**Table of Contents**


A. General Considerations S3

B. Synthesis and Characterization S5

C. Supporting Data S8

D. Olefin Epoxidation S20

E. C–H Oxidation S19

F. Recycling Studies S21

G. Control Experiments with $CoCl_2$ S22

H. Kinetic Isotope Effect Data S23

I. Epoxidation Diastereoselectivity S25

J. X-Ray Crystallographic Data S26

K. References S30

## A. General Considerations

**Materials** All the commercial reagents and solvents (ACS reagent grade) were used as received. Tetralin (**2a**), potassium *tert*-butoxide, mercury(II) chloride, cyclohexene, *cis*-2-hexene (*cis*-**1e**), *cis*-*β*-methylstyrene (*cis*-**1d**), and anhydrous *N*,*N*-dimethylformamide (DMF) were obtained from Sigma Aldrich. Norbornene was obtained from Alfa Aesar. Silica gel (0.06–0.20 mm, 60 Å for column chromatography) and indane were obtained from Acros Organics. Potassium permanganate, anhydrous iron(II) chloride and zinc dust were obtained from Strem Chemicals. $CoCl_2 \cdot 6H_2O$ was obtained from Fisher Scientific; sodium azide was obtained from BeanTown Chemical; ammonium chloride, isobutyraldehyde, cycloheptene, fluorene, and 4-cyano benzoic acid were obtained from TCI; and, ammonium formate and *meta*-chloroperoxybenzoic acid were obtained from Oakwood Chemical. NMR solvents were purchased from Cambridge Isotope Laboratories and stored over 4 Å molecular sieves. GC standards for *trans*-β-methylstyrene oxide (*trans*-**3d**), *cis*-β-methylstyrene oxide (*cis*-**3d**), *trans*-2,3-epoxyhexane (*trans*-**3e**), and *cis*-2,3-epoxyhexane (*cis*-**3e**) were prepared according to literature methods.[1]

**Characterization Details** NMR spectra were recorded on Bruker Avance NEO 400 NMR or Varian 400 MHz Inova NMR operating at 400.09 MHz for $^1H$ acquisitions and were referenced against solvent signal: $CD_3OD$ (3.28 ppm, $^1H$).[3] $^1H$ NMR data are reported as follows: chemical shift (δ, ppm), multiplicity (s (singlet), d (doublet), t (triplet), m (multiplet), br (broad), integration. ATR-IR spectra were recorded on a Shimadzu FTIR/IRAffinity-1 spectrometer and were determined as the average of 64 scans. FE-SEM imaging was performed on a JSM7500 (RRID: SCR022202) FE-SEM with a field emission at 5 kV.

**Powder X-ray Diffraction** PXRD measurements were carried out on a Bruker D8 Advance Eco X-ray diffractometer (Cu Kα, 1.5418 Å; 40 kV, 25 mA) fitted with a LynxEye detector. The angular range was measured from 4.00 to 25.00° (2θ) with steps of 0.010° and a measurement time of 0.3 second per step. Simulated PXRD patterns were calculated using Mercury 3.9.

**Single Crystal X-ray Diffraction** An as-synthesized purple plate-shaped single crystal of TAM-3 was used for single crystal analysis. The selected crystal (0.07 × 0.06 × 0.04 $mm^3$) was mounted on a MITIGEN holder on a Bruker QUEST diffractometer. The X-ray radiation employed was generated from a Mo-sealed X-ray tube (Kα = 0.70173 Å with a potential of 40 kV and a current of 40 mA). The crystal was maintained at T = 100 K during data collection. The structure was solved with the ShelXT 2018/2 solution program using dual methods and by using Olex2 1.5 as the graphical interface. The model was refined with ShelXL 2018/3 using full matrix least squares minimization on $F^2$. The data was found to be from a weakly diffracting crystal with no reflections above 42 degrees two-theta; examination of other crystals obtained from the described synthesis afforded similarly weak diffraction. Disordered solvent molecules were masked using Olex2 and the R-factors are thus high.

**UV-vis Spectroscopy** Solution-phase UV-vis spectra were recorded on a Shimadzu 2501PC spectrometer with DH UV-vis-NIR light source (190–1100 nm). Solution-phase spectra were

blanked against the appropriate solvent. Solid-state UV-vis spectra were recorded on a Hitachi U-4100 spectrophotometer (341-F) with UV-vis-NIR light source (175–2600 nm) and blanked against the appropriate quartz slide.

**Thermogravimetric Analysis** TGA measurements were conducted on a METTLER TOLEDO TGA/DSC 1, with as-synthesized TAM-3 crystals that were partially activated by keeping it under vacuum for 24 h at 25 °C. The temperature was increased from 25 °C to 120 °C at a ramp rate of 5 °C/min and held at that for 60 min under vacuum to complete the activation. The temperature was allowed to decrease to 25 °C at a ramp rate of -5 °C/min, and then raised to 800 °C, at a ramp rate of 5 °C/min.

**Mass Spectroscopy** Atmospheric pressure chemical ionization mass spectrometry (APCI-MS) experiments were performed using a Thermo Scientific Q Exactive Focus operating in positive mode. Samples were injected into a 10 μL loop and methanol was used as a mobile phase at a flow rate of 500 μL/min. The mass resolution was tuned to 70000 FWHM at m/z 200. Exactive Series 2.11 /Xcalibur 4.02.47 software was used for data acquisition and processing.

## B. Synthesis and Characterization

### Synthesis of 4-(*2H*-tetrazol-5-yl)benzoic acid

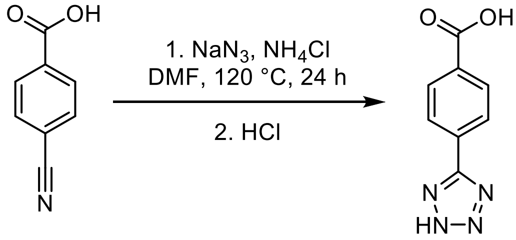

4-(*2H*-tetrazol-5-yl)benzoic acid ($H_2TBA$) was synthesized according to the following modification of literature methods.[2] A 20-mL round-bottomed flask was charged with sodium azide (325 mg, 5.00 mmol, 1.00 equiv), ammonium chloride (267 mg, 5.00 mmol, 1.00 equiv) and anhydrous DMF (5 mL). The mixture was stirred for 30 min at 25 °C and 4-cyano benzoic acid (736 mg, 5.00 mmol, 1.00 equiv) was slowly added. The resulting mixture was heated to reflux for 24 h. The flask was allowed to cool to 25 °C and 10 mL HCl (1 M) was added dropwise with stirring. The white precipitate was filtered and washed with distilled water (3 × 5 mL) and diethyl ether (3 × 5 mL). The resulting white powder was dried under vacuum at 50 °C to yield $H_2TBA$ (92%, 875 mg). $^1H$ NMR ($\delta$, 23 °C, 400 MHz, DMSO-$d_6$): δ 8.16 (m, 4H), 13.28 (br s, 1H).

### Synthesis of TAM-3

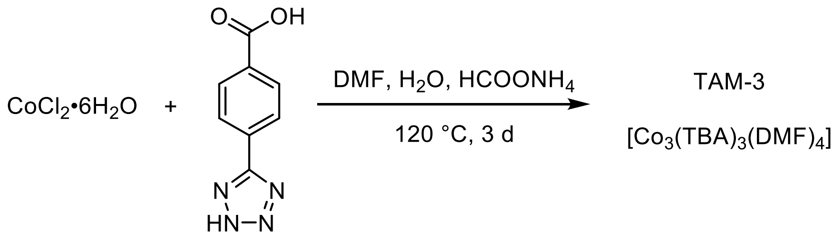

A 20-mL scintillation vial was charged with $CoCl_2 \cdot 6H_2O$ (50.0 mg, 0.210 mmol, 1.00 equiv), $H_2TBA$ (40.0 mg, 0.210 mmol, 1.00 equiv), and ammonium formate (8.00 mg, 0.127 mmol, 0.605 equiv). DMF (1.56 mL) and $H_2O$ (143 μL) were added to the reaction vessel and the resulting mixture was agitated by sonication until a dark blue solution was obtained. The reaction vessel was placed in an oven pre-heated at 120 °C for 3 d. The reaction vial was removed from the oven and the hot supernatant was immediately decanted. Fresh DMF (2 mL) was added, and the resulting mixture was stored at 23 °C for 16 h before the DMF was decanted. Additional DMF was added and the soaking (16 h) / decanting cycle was repeated three times, at which point the decanted DMF was colorless. Dark purple crystals of formula $[Co_3(TBA)_3(DMF)_4]$ were obtained. DMF was replaced with DCE (2 mL) and the crystals were soaked for 24 h. The solvent was decanted and fresh DCE (2 mL) was added. After 24 h all

the solvent was decanted and the MOF was allowed to air dry to afford TAM-3 as a purple powder (43 mg, 59% yield). The crystals were characterized by PXRD, IR, TGA and DR-UV-vis spectroscopy.

**Synthesis of *$d_4$*-tetralin (*$d_4$*-2a)**

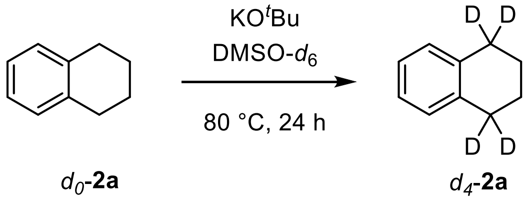


*$d_4$*-Tetralin was prepared according to the following modification of literature methods.[3] A 25-mL Schlenk flask was charged with 1,2,3,4-tetrahydronaphthalene (661 mg, 5.00 mmol, 1.00 equiv), potassium *tert*-butoxide (113 mg, 1.00 mmol, 0.10 equiv), and DMSO-*$d_6$* (5 mL) under an $N_2$ atmosphere. The resulting solution was heated at 80 °C for 24 h. The solution was allowed to cool to 23 °C and quenched with water (25 mL). The organics were extracted with diethyl ether (50 mL × 3) and washed with water (50 mL × 3). The combined ether extract was dried over anhydrous $Na_2SO_4$ and concentrated under vacuum. The obtained residue was re-subjected to the above-described procedure a second time. The resulting liquid was passed through a short plug of silica with hexanes as the eluent and concentrated under vacuum. The obtained liquid was then distilled under reduced pressure to afford *$d_4$*-**2a** as a colorless oil (511 mg, 75% yield, 97% D-incorporation). $^1$H NMR ($\delta$, 23 °C, 400 MHz, $CDCl_3$): δ 7.11–7.05 (m, 4H), 1.79 (s, 4H).

**Synthesis of *$d_2$*- tetralin (*$d_2$*-2a)**

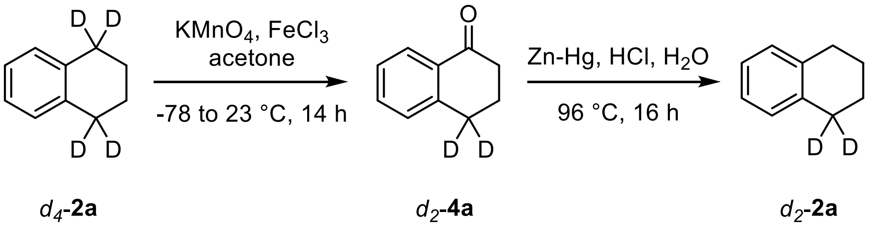


*$d_2$*-Tetralin was prepared according to the following modification of literature methods.[3] A 100-mL round-bottom flask was charged with *$d_4$*-**2a** (272 mg, 2.00 mmol, 1.00 equiv) in acetone (20 mL) was cooled to −78 °C with a dry ice / acetone bath. $KMnO_4$ (1.58 g, 10.0 mmol, 5.00 equiv) and $FeCl_3$ (0.40 g, 2.50 mmol, 1.25 equiv) were added to the solution and stirred at −78 °C for 2 h. The mixture was allowed to warm up to 23 °and stirred for 12 h. $CH_2Cl_2$ (50 mL) was added and stirred for 1 h. The solids were separated by filtration and washed with $CH_2Cl_2$ (50 mL × 3). The combined filtrate was concentrated under reduced pressure and purified by silica gel chromatography (20% ethyl acetate in hexanes) to yield

ketone *$d_2$*-**4a** as a pale-yellow oil (224 mg, 83% yield). $^1$H NMR ($\delta$, 23 °C, 400 MHz, $CDCl_3$): δ 8.04 (ddd, $J$ = 7.8, 1.5, 0.5 Hz, 1H), 7.47 (td, $J$ = 7.5, 1.5 Hz, 1H), 7.33–7.29 (m, 1H), 7.27–7.24 (m, 1H), 2.68–2.64 (m, 2H), 2.13 (t, $J$ = 6.5 Hz, 2H).

A 25-mL round-bottom flask was charged with Zn dust (495 mg, 7.74 mmol, 5.21 equiv) and $HgCl_2$ (44 mg, 0.162 mmol, 0.109 equiv). $H_2O$ (1.00 mL) and conc. HCl (100 µL) were added, and the mixture was stirred at 23 °C for 5 min. The liquid phase was decanted from the activated Zn-Hg. Ketone *$d_2$*-**4a**, (220 mg, 1.48 mmol, 1.00 equiv), $H_2O$ (1.00 mL), and conc. HCl (300 µL) were added, and the mixture was heated at 96 °C for 16 h. The reaction was cooled to 23 °C and extracted with diethyl ether (10 mL × 3). The combined organic layer was concentrated under vacuum and purified by silica gel chromatography with hexanes. The obtained liquid was distilled under reduced pressure to afford *$d_2$*-tetralin as a colorless oil (120 mg, 60% yield). $^1$H NMR ($\delta$, 23 °C, 500 MHz, $CDCl_3$): δ 7.11–7.06 (m, 4H), 2.79 (dd, $J$ = 7.7, 3.9 Hz, 2H), 1.81 (t, $J$ = 4.3 Hz, 4H).

## C. Supporting Data

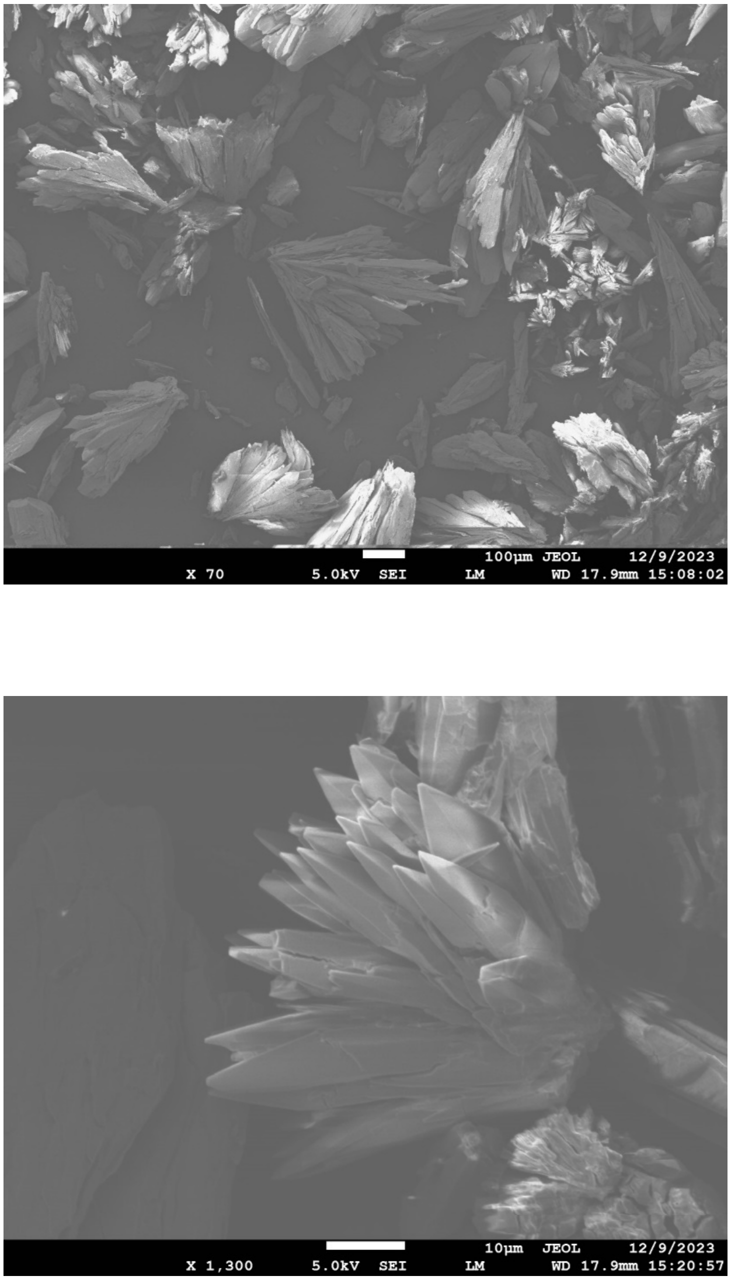


**Figure S1**. FE-SEM images of as-synthesized crystals of TAM-3 (top: 100 µm scale, bottom: 10 µm scale).

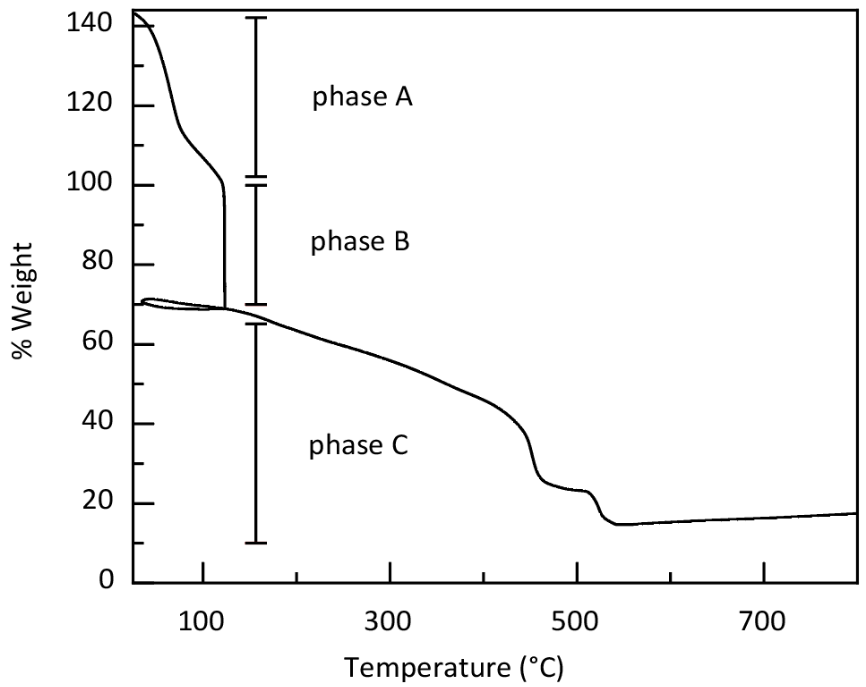


**Figure S2**. Thermogravimetric analysis of TAM-3 (Phase A: loss of unbound DMF molecules as the temperature increased to 120 °C. Phase B: loss of 30% weight attributed to the bound DMF molecules as the MOF was held at 120 °C for 60 min under vacuum, at the end of which no further change in weight was observed. Phase C: temperature increased to 800 °C at a ramp rate of 5 °C/min).

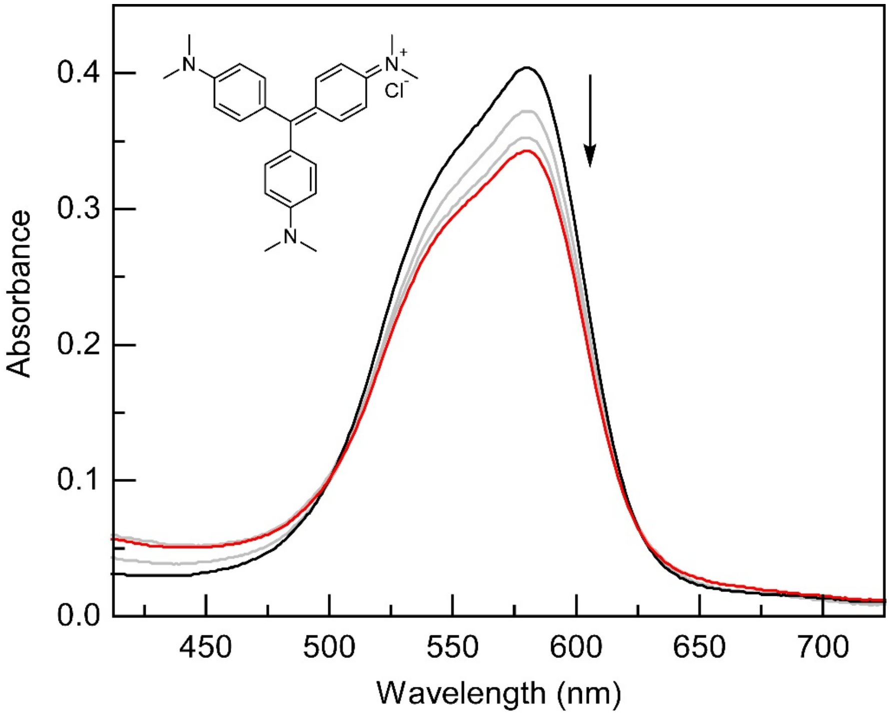


**Figure S3**. UV-vis spectra obtained periodically over 24 h for the supernatant of a sample of TAM-3 (1 mg) exposed to an acetone solution of crystal violet (5 μM, 3 mL).

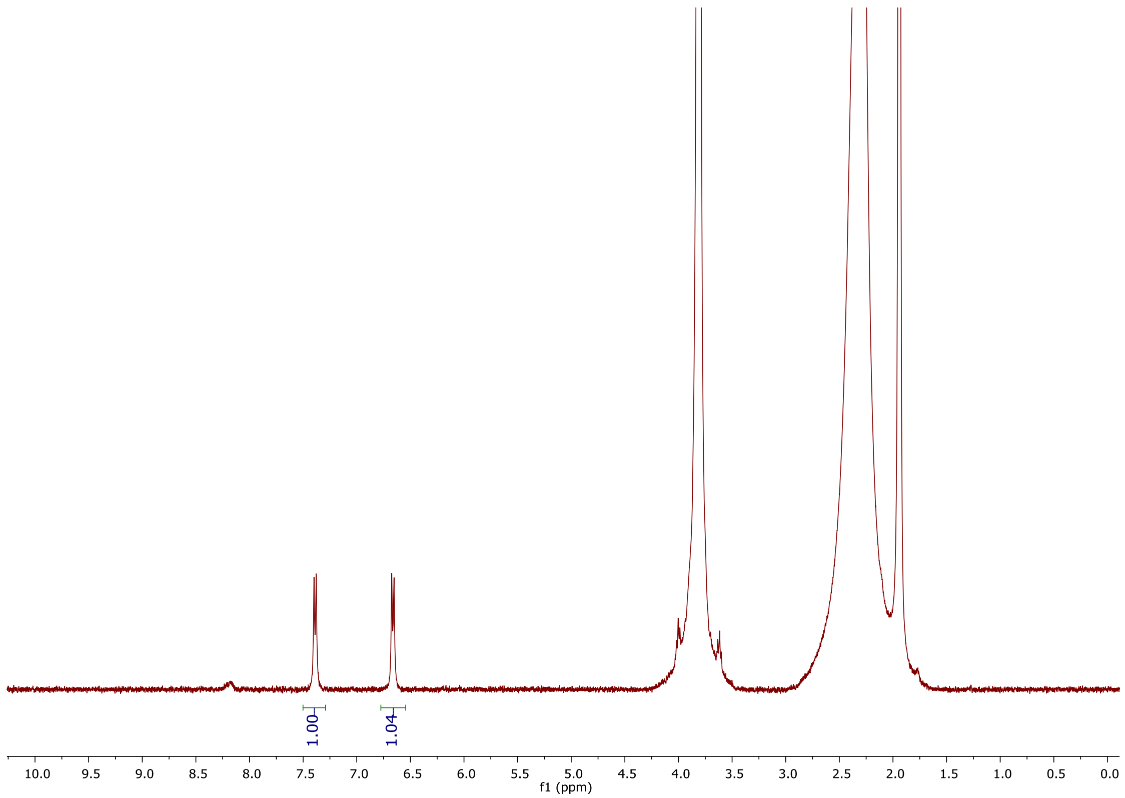


**Figure S4**. $^1$H NMR spectrum showing expulsion of 4-aminobenzonitrile from TAM-3 by $CD_3CN$. Crystals of TAM-3 was soaked in a solution of 4-aminobenzonitrile (10 mg) in DCE (2 mL) for 6 h. After this time the supernatant was decanted. This process was repeated two more times. After decanting the supernatant, DCE (2 mL) was added and kept for 6 h. Solvent was decanted and the DCE soaking was repeated two more times. The supernatant was decanted. The air-dried crystals were transferred to an NMR tube and $CD_3CN$ was added. Characteristic peaks for 4-aminobenzonitrile were observed in the $^1$H NMR spectrum. $^1$H NMR ($\delta$, 23 °C, 500 MHz, $CDC_3CN$): δ 7.39 (d, $J$ = 8.4 Hz, 1H), 6.66 (d, $J$ = 8.3 Hz, 1H). Analytic data is in accordance with literature data.[4]

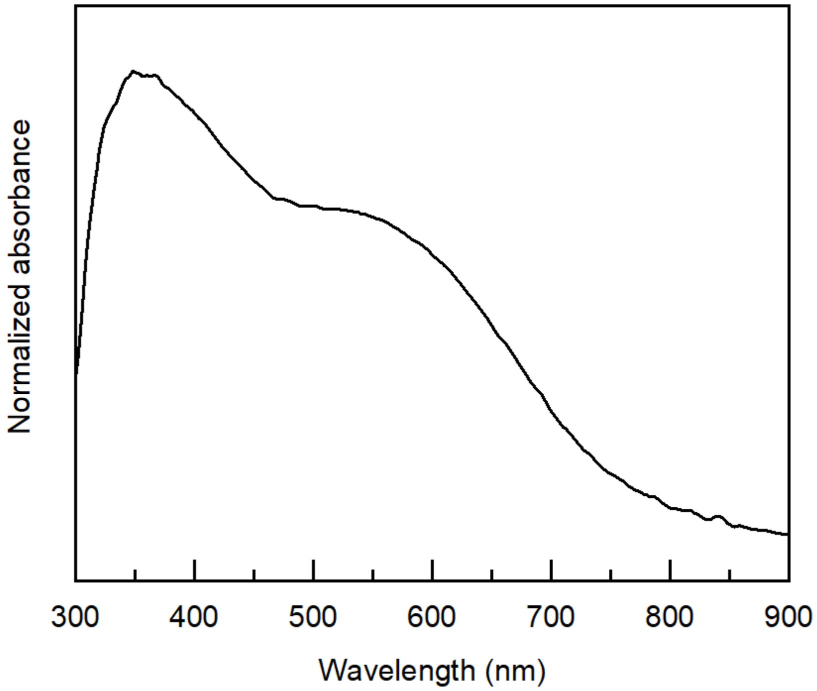


**Figure S5**. Diffuse reflectance UV-vis spectrum of TAM-3 after reaction with $O_2$ in the presence of isobutyraldehyde.

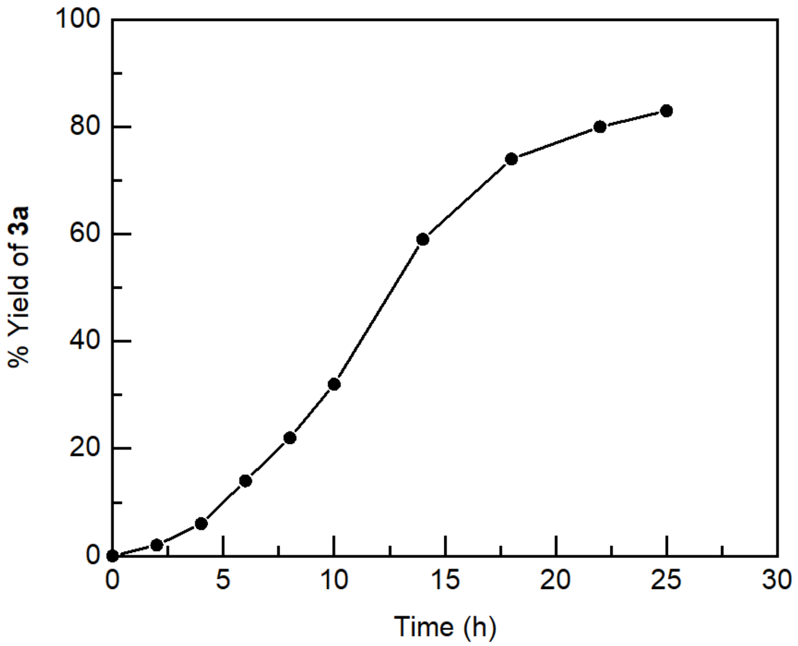


**Figure S6**. Percent yield of **3a** over time from the epoxidation reaction of **1a** under standard conditions, showing a sigmoidal curve with an induction period. A 20-mL scintillation vial was charged with cyclohexene (25.3 µL, 0.250 mmol, 1.00 equiv) and DCE (2 mL). TAM-3 (5.16 mg, 5.00 µmol, 2.00 mol%) was added and the vial was fitted with a rubber septum and an $O_2$-filled balloon. Isobutyraldehyde (45.6 µL, 0.500 mmol, 2.00 equiv) was added, and the mixture was stirred at 23 °C. Small aliquots were taken out from the reaction mixture in certain time intervals and the formation of was analyzed by 1H NMR spectroscopy in $CDCl_3$.

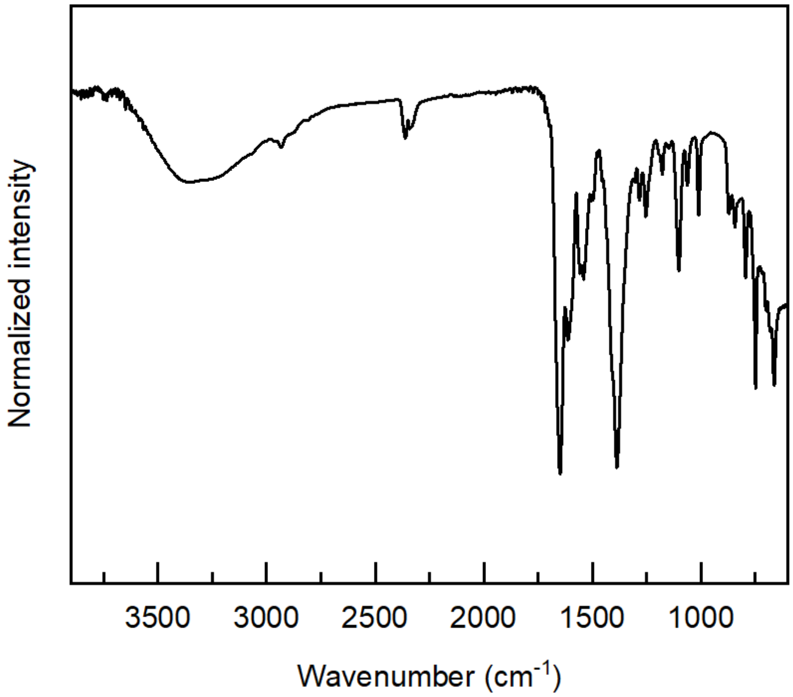


**Figure S7.** IR spectrum of TAM-3, solvent exchanged with DCE and air dried, showing characteristic NNN, NCN, and CNN stretches attributed to the tetrazole fragment in the ligand at 1612, 1558, 1498, and 1407 $cm^{-1}$, and stretches for Co-bound DMF ligand at 2930, 1649, 1386, and 1101 $cm^{-1}$.

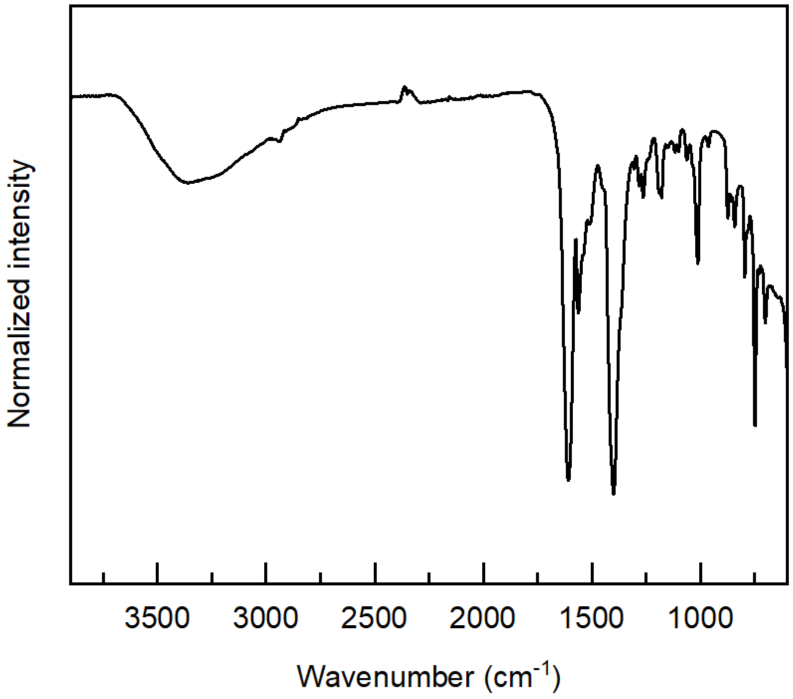


**Figure S8.** IR spectrum of TAM-3, solvent exchanged with DCE and activated under vacuum at 25 °C for 6 h.

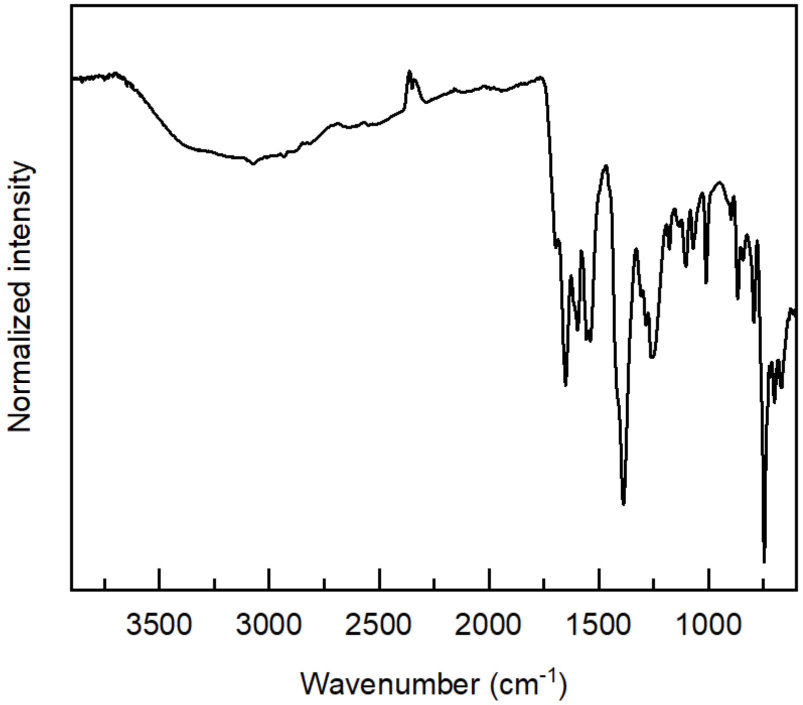


**Figure S9.** IR spectrum of TAM-3, solvent exchanged with DCE and treated with a dilute DCE solution of excess mCPBA.

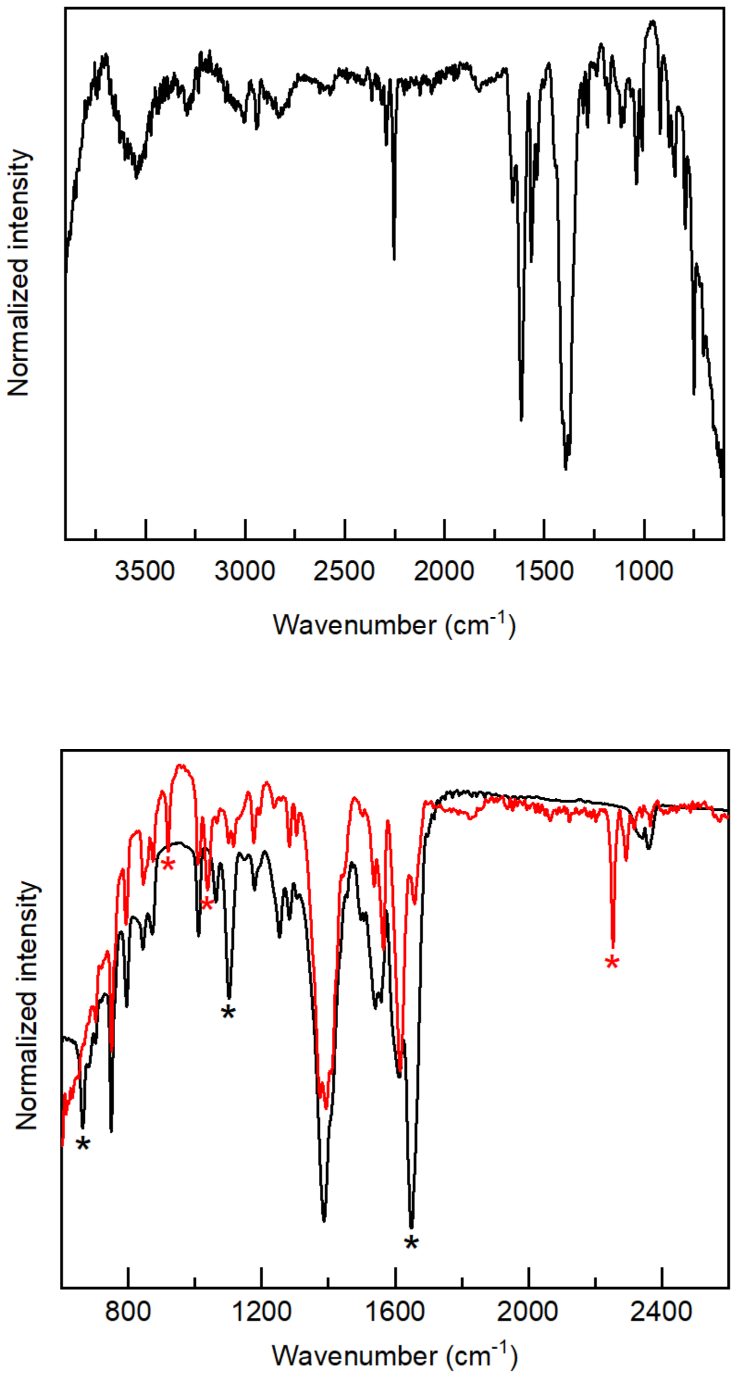


**Figure S10.** IR spectrum of TAM-3, solvent exchanged with acetonitrile (top), and overlay with the IR spectrum of TAM-3 (bottom): stretches for DMF are marked with (*) and that of acetonitrile are marked with (*).

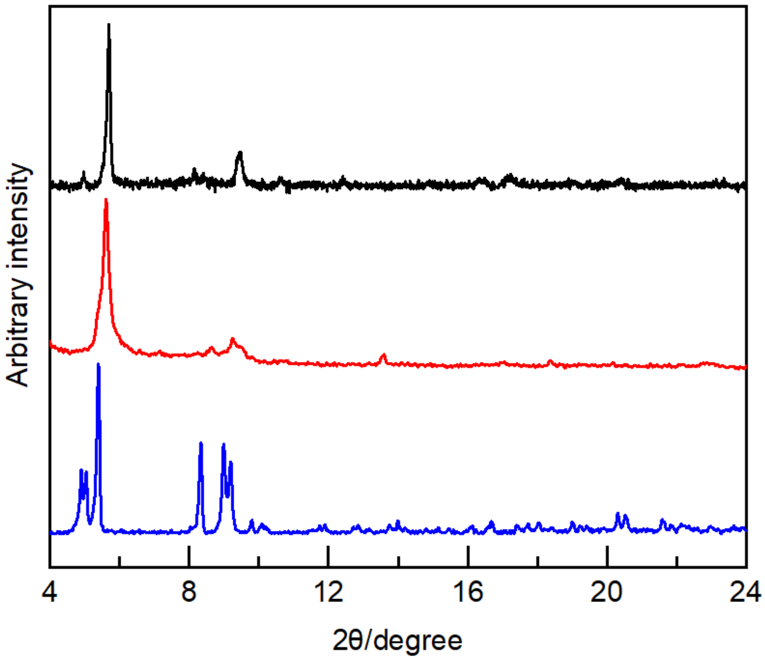


**Figure S11.** PXRD pattern of TAM-3 after a cyclohexene epoxidation run of 24 h at 23 °C (three parallel reactions were run and the combined MOF was used for PXRD analysis) (—), treated with a dilute DCE solution of excess mCPBA (—), and as synthesized (—)

## D. Olefin Epoxidation

A 20-mL scintillation vial was charged with the appropriate olefin (0.250 mmol, 1.00 equiv) and DCE (2 mL). TAM-3 (5.16 mg, 5.00 µmol, 2.00 mol%) was added and the vial was fitted with a rubber septum and an $O_2$-filled balloon. Isobutyraldehyde (45.6 µL, 0.500 mmol, 2.00 equiv) was added, and the mixture was stirred at 23 °C for 24 h. The reaction mixture was filtered through a Celite plug. The organics were washed with 2 mL deionized water twice and dried over $Na_2SO_4$. Mesitylene was added as an internal standard and the yield was calculated from the $^1$H NMR of the crude reaction mixture.

**Cyclohexene oxide (3a)** Obtained in 92% yield. $^1$H NMR (*δ*, 23 °C, 500 MHz, $CDCl_3$): δ 3.11 (dt, *J* = 2.4, 1.1 Hz, 2H), 1.99–1.89 (m, 2H), 1.85–1.74 (m, 2H), 1.45–1.35 (m, 2H), 1.24–1.19 (m, 2H). Analytic data is in accordance with literature data.[5]

**Cycloheptene oxide (3b)** Obtained in 90% yield. $^1$H NMR (*δ*, 23 °C, 500 MHz, $CDCl_3$): δ 3.12–3.03 (m, 2H), 1.99–1.86 (m, 4H), 1.64–1.57 (m, 1H), 1.52–1.40 (m, 4H), 1.22–1.16 (m, 1H). Analytic data is in accordance with literature data.[6]

**Norbornene oxide (3c)** Obtained in 94% yield. $^1$H NMR (*δ*, 23 °C, 500 MHz, $CDCl_3$): δ 3.03 (s, 2H), 2.41 (br s, 2H), 1.42–1.47 (m, 2H), 1.25–1.30 (m, 1H), 1.15–1.19 (m, 2H), 0.67 (d, *J* = 9.6 Hz, 1H). Analytic data is in accordance with literature data.[7]

## E. C–H Oxidation

A 20-mL scintillation vial was charged with the appropriate substrate (0.250 mmol, 1.00 equiv) and DCE (2 mL). TAM-3 (5.16 mg, 5.00 μmol, 2.00 mol%) was added and the vial was fitted with a rubber septum and an $O_2$-filled balloon. Isobutyraldehyde (45.6 μL, 0.500 mmol, 2.00 equiv) was added, and the mixture was stirred at 23 °C for 16 h. A second portion of isobutyraldehyde (45.6 μL, 0.500 mmol, 2.00 equiv) was added, and the mixture was stirred at 23 °C for 16 h. The $O_2$ balloons were refilled and a third portion of isobutyraldehyde (45.6 μL, 0.500 mmol, 2.00 equiv) was added, and the mixture was stirred at 23 °C for additional 16 h. The reaction mixture was filtered through a Celite plug. The organics were washed with 2 mL deionized water twice and dried over $Na_2SO_4$. Mesitylene was added as an internal standard and the yield was calculated from the $^1$H NMR of the crude reaction mixture.

**1-Tetralone (4a)** Obtained in 78% yield. $^1$H NMR ($\delta$, 23 °C, 500 MHz, $CDCl_3$): δ 8.03 (dd, *J* = 7.8, 1.5 Hz, 1H), 7.47 (td, *J* = 7.5, 1.5 Hz, 1H), 7.34–7.28 (m, 1H), 7.25 (d, *J* = 7.5 Hz, 1H), 2.97 (t, *J* = 6.1 Hz, 2H), 2.66 (dd, *J* = 7.3, 5.8 Hz, 2H), 2.14 (p, *J* = 6.5 Hz, 2H). Analytic data is in accordance with literature data.[8]

**1-Indanone (4b)** Obtained in 67% yield. $^1$H NMR ($\delta$, 23 °C, 500 MHz, $CDCl_3$): δ 7.76 (d, *J* = 7.7 Hz, 1H), 7.62–7.55 (m, 1H), 7.48 (dq, *J* = 7.8, 0.9 Hz, 1H), 7.40–7.34 (m, 1H), 3.18–3.11 (m, 2H), 2.72–2.66 (m, 2H). Analytic data is in accordance with literature data.[8]

**Fluorenone (4c)** Obtained in 40% yield. $^1$H NMR ($\delta$, 23 °C, 500 MHz, $CDCl_3$): δ 7.66 (dd, *J* = 7.4, 1.0 Hz, 1H), 7.55–7.44 (m, 2H), 7.32–7.27 (m, 1H). Analytic data is in accordance with literature data.[9]

**1-Adamantanol (4d)** Obtained in 32% yield. $^1$H NMR ($\delta$, 23 °C, 500 MHz, $CDCl_3$): δ 2.13 (s, 3H), 1.71 (d, *J* = 2.9 Hz, 6H), 1.61 (q, *J* = 12.3 Hz, 6H). Analytic data is in accordance with literature data.[10]

### F. Recycling Studies

A 20-mL scintillation vial was charged with cyclohexene (**1a**) (25.3 μL, 0.250 mmol, 1.00 equiv) and DCE (2 mL). TAM-3 (5.16 mg, 5.00 μmol, 2.00 mol%) was added and the vial was fitted with a rubber septum and an $O_2$-filled balloon. Isobutyraldehyde (45.6 μL, 0.500 mmol, 2.00 equiv) was added, and the mixture was stirred at 23 °C for 24 h. The solution was decanted, DCE (0.5 mL) was added and kept for 6 h. The solution was decanted and combined with previous DCE fraction. Another portion of fresh DCE (0.5 mL) was added and kept for 6 h. The solution was decanted and combined with previous DCE fractions. This crude mixture was analyzed by $^1H$ NMR with $CDCl_3$ as the solvent. **3a** was formed in 87% yield.

The vial with recycled TAM-3 was charged with cyclohexene (**1a**) (25.3 μL, 0.250 mmol, 1.00 equiv) and DCE (2 mL). Isobutyraldehyde (45.6 μL, 0.500 mmol, 2.00 equiv) was added, and the mixture was stirred at 23 °C for 24 h. After which the reaction mixture was analyzed following the same procedure as above, and **3a** was found to form in 78% yield.

A third run was carried out with the recycled TAM-3 from previous reaction, following the same procedure, and **3a** was found to form in 90% yield.

## G. Control Experiments with $CoCl_2$

### Epoxidation of 1a to 3a

A 20-mL scintillation vial was charged with cyclohexene (**1a**) (25.3 μL, 0.250 mmol, 1.00 equiv) and DCE (2 mL). $CoCl_2\cdot 6H_2O$ (1.19 mg, 2.00 mol%) was added and the vial was fitted with a rubber septum and an $O_2$-filled balloon. Isobutyraldehyde (45.6 μL, 0.500 mmol, 2.00 equiv) was added, and the mixture was stirred at 23 °C for 24 h. Mesitylene was added as an internal standard and the yield was calculated from the $^1H$ NMR of the crude reaction mixture. **3a** was found to form in 89% yield.

### Oxidation of 2a to 4a

A 20-mL scintillation vial was charged with tetralin (**2a**) (34.1 μL, 0.250 mmol, 1.00 equiv) and DCE (2 mL). $CoCl_2\cdot 6H_2O$ (1.19 mg, 2.00 mol%) was added and the vial was fitted with a rubber septum and an $O_2$-filled balloon. Isobutyraldehyde (45.6 μL, 0.500 mmol, 2.00 equiv) was added, and the mixture was stirred at 23 °C for 16 h. A second portion of isobutyraldehyde (45.6 μL, 0.500 mmol, 2.00 equiv) was added, and the mixture was stirred at 23 °C for 16 h. The $O_2$ balloons were refilled and a third portion of isobutyraldehyde (45.6 μL, 0.500 mmol, 2.00 equiv) was added, and the mixture was stirred at 23 °C for additional 16 h. Mesitylene was added as an internal standard and the yield was calculated from the $^1H$ NMR of the crude reaction mixture. **4a** was found to form in 66% yield.

## H. Kinetic Isotope Effect Data

### Determination of Intramolecular KIEs

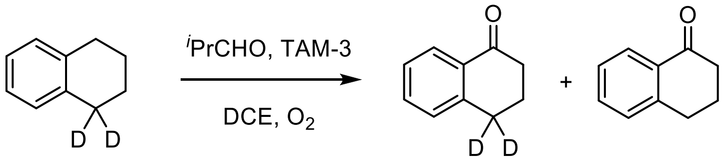


A 20-mL scintillation vial was charged with $d_2$-**2a** (16.1 mg, 0.120 mmol, 1.00 equiv) and DCE (2 mL). TAM-3 (3.10 mg, 3.00 µmol, 2.50 mol%) was added and the vial was fitted with a rubber septum and a balloon filled with $O_2$. Isobutyraldehyde (7.62 µL, 84.0 µmol, 0.700 equiv) was added and the mixture was stirred at 23 °C for 24 h. The crude mixture was filtered through a short Celite plug and analyzed by mass spectroscopy using APCI-MS. Integration of the MS signal at m/z= 149.0930 (plus the natural abundance signal at 150.0963) versus m/z = 147.0804 (plus the natural abundance signal at 148.0838) provided the ratio of C–D versus C–H cleavage. An example of the MS data that was used for this analysis is reproduced in Figure S8 below.

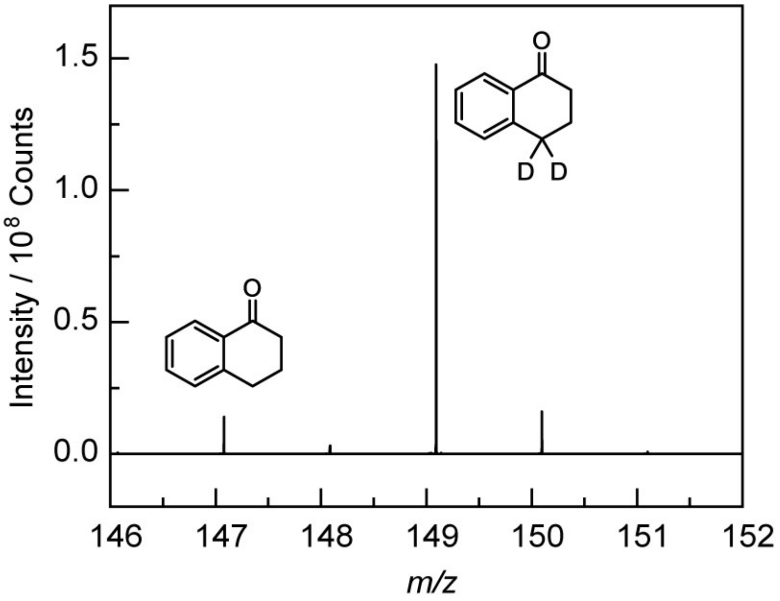


**Figure S12**. APCI-MS data obtained following oxidation of $d_2$-**2a** in the presence of TAM-3.

**Intermolecular KIE Determination**

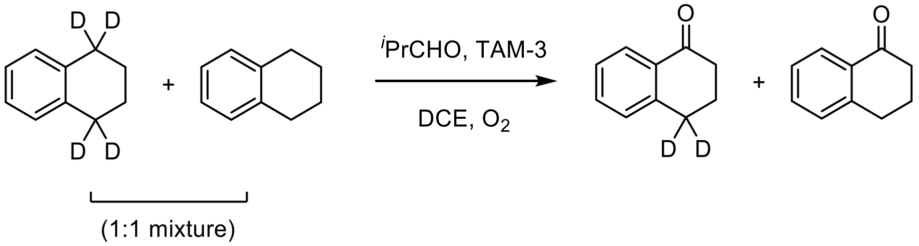


A 20-mL scintillation vial was charged with *d₄*-**2a** (1.00 mL) and *d₀*-**2a** (1.00 mL). A second 20-mL scintiallatoin vial was charged with this mixture (100 µL, 0.734 mmol, 4.89 equiv) and DCE (2 mL). TAM-3 (3.10 mg, 3.00 µmol, 0.0200 equiv) was added and the vial was fitted with a rubber septum and a balloon filled with isobutyraldehyde (13.7 µL, 0.150 mmol, 1.00 equiv) was added and the mixture was stirred at 23 °C for 24 h. Another portion of isobutyraldehyde (13.7 µL, 0.150 mmol, 1.00 equiv) was added and it was stirred at 23 °C for 24 h. The crude mixture was then filtered through a short Celite plug, analyzed by $^1$H NMR with $CDCl_3$ as solvent. Reactions where the total yield of the two ketones was below 10% were analyzed by mass spectroscopy using APCI-MS. Integration of the MS signal at m/z = 147.0804 (plus the natural abundance signal at 148.0838) versus m/z= 149.0930 (plus the natural abundance signal at 150.0963) provided the ratio of C–H versus C–D cleavage. An example of the MS data that was used for this analysis is reproduced in Figure S9 below.

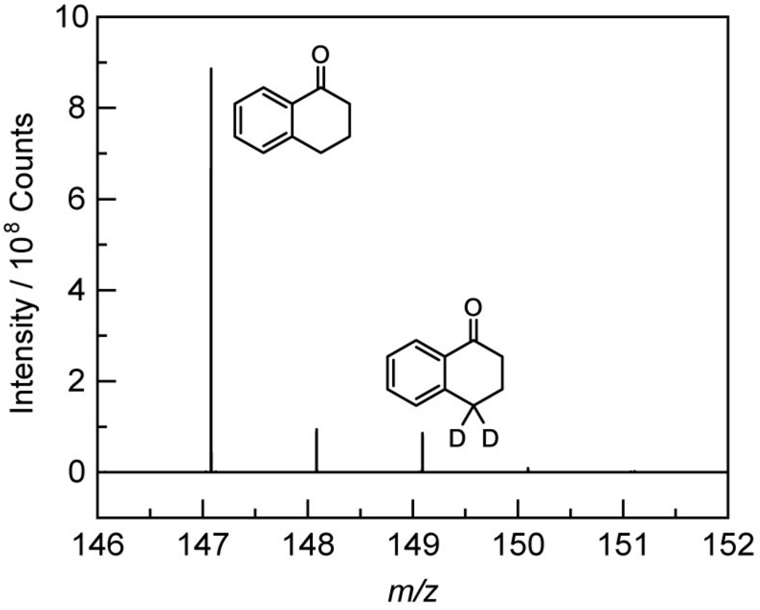


**Figure S13**. APCI-MS data obtained following the oxidation of a 1 : 1 mixture of $d_4$-**2a** and $d_0$-**2a** in the presence of TAM-3.

## I. Epoxidation Diastereoselectivity

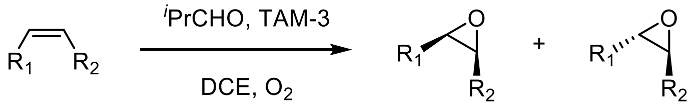


**General Procedure** A 20-mL scintillation vial was charged with the appropriate olefin (0.250 mmol, 1.00 equiv) and DCE (2 mL). TAM-3 (5.16 mg, 5.00 µmol, 2.00 mol%) was added and the vial was fitted with a rubber septum and an $O_2$-filled balloon. Isobutyraldehyde (45.6 µL, 0.500 mmol, 2.00 equiv) was added, and the mixture was stirred at 23 °C for 24 h. The reaction mixture was filtered through a Celite plug and the product distribution was analyzed by gas chromatography. The retention times of the epoxide isomers were confirmed by co-injection of authentic standards.

## J. X-Ray Crystallographic Data

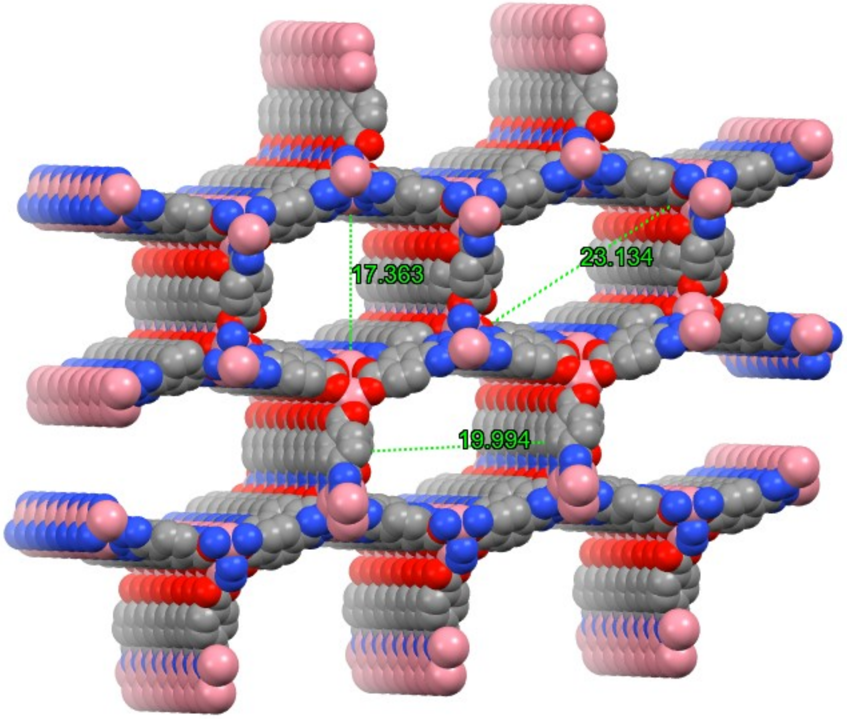


**Figure S14**. Space-filling model of TAM-3 (DMF molecules omitted for clarity) showing hexagonal channels with two diagonals of 17 Å and 23 Å, and width of 20 Å.

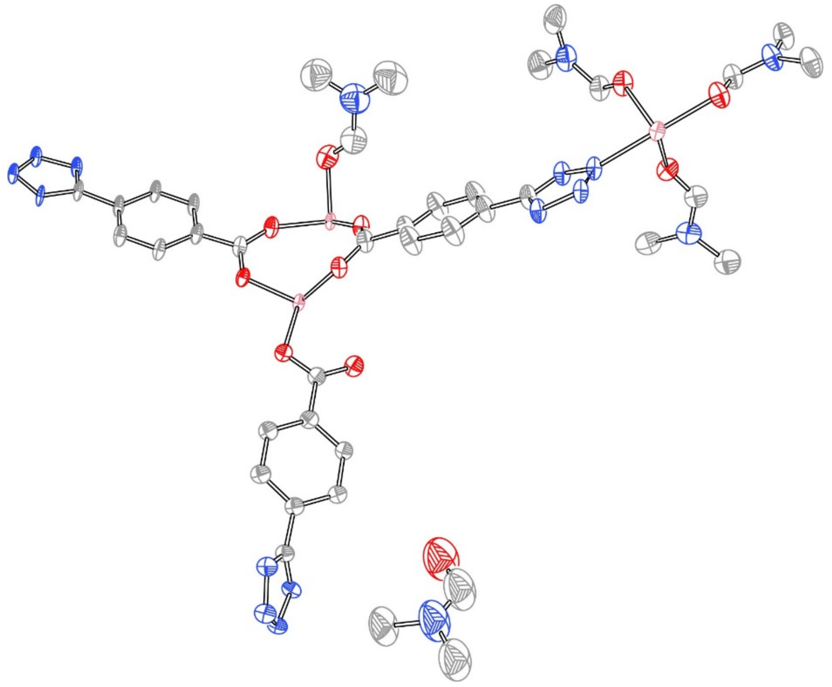

**Figure S15**. Displacement ellipsoid plot of the asymmetric unit of TAM-3 plotted at 50% probability. H-atoms are removed for clarity.

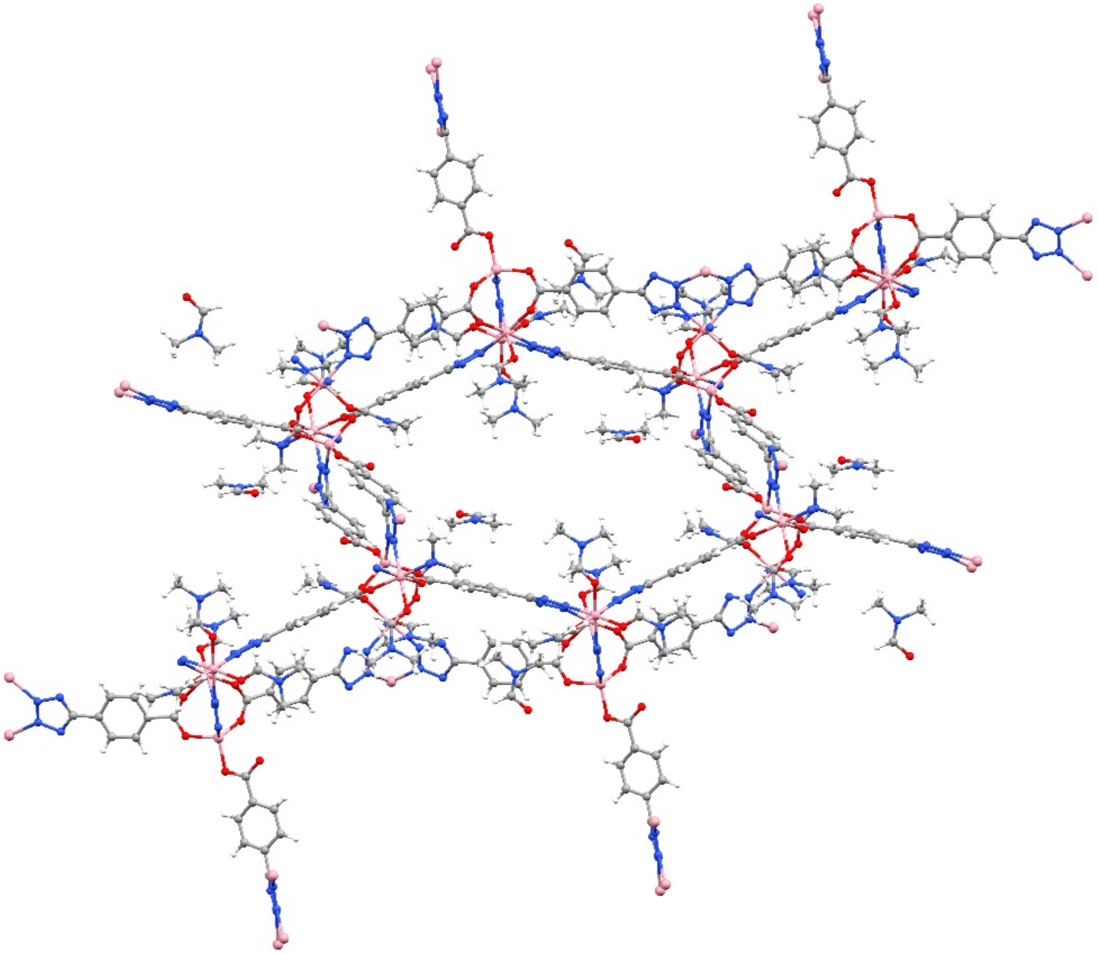

**Figure S16**. Hexagonal pore structure of TAM-3 shown by packing the asymmetric unit.

**Table S1.** X-ray experimental details of TAM-3 (CCDC 2421775).

| | |
|---|---|
| **Crystal data** | |
| **Chemical formula** | $C_{36}H_{40}Co_3N_{16}O_{10}·C_3H_7NO·3.85[C_3H_7NO]$ |
| $M_r$ | 1388.14 |
| **Crystal system, space group** | Monoclinic, $P2_1/n$ |
| **Temperature (K)** | 100 |
| ***a*, *b*, *c* (Å)** | 21.854(2), 11.1155(8), 30.817(2) |
| **β (°)** | 96.776(2) |
| ***V* (Å³)** | 7434(1) |
| ***Z*** | 4 |
| **Radiation type** | Mo *K*α |
| **μ (mm⁻¹)** | 0.73 |
| **Crystal size (mm)** | 0.07 × 0.06 × 0.04 |
| **Data collection** | |
| **Diffractometer** | Bruker QUEST |
| **Absorption correction** | Multi-scan *SADABS2016*/2 (Bruker, 2016/2) was used for absorption correction. wR2(int) was 0.0897 before and 0.0696 after correction. The Ratio of minimum to maximum transmission is 0.8045. The λ/2 correction factor is not present. |
| $T_{min}$, $T_{max}$ | 0.599, 0.745 |
| **No. of measured, independent and observed [$I > 2\sigma(I)$] reflections** | 63786, 8010, 7138 |
| $R_{int}$ | 0.073 |
| $\theta_{max}$ (°) | 21.0 |
| $(\sin \theta/\lambda)_{max}$ (Å⁻¹) | 0.505 |
| **Refinement** | |
| $R[F^2 > 2\sigma(F^2)]$, $wR(F^2)$, $S$ | 0.117, 0.287, 1.12 |
| **No. of reflections** | 8010 |
| **No. of parameters** | 641 |
| **No. of restraints** | 921 |
| **H-atom treatment** | H-atom parameters constrained |
| | $w = 1/[\sigma^2(F_o^2) + (0.0875P)^2 + 177.334P]$ where $P = (F_o^2 + 2F_c^2)/3$ |
| $\Delta\rho_{max}$, $\Delta\rho_{min}$ (e Å⁻³) | 0.88, −1.06 |

## K. References


1. Mulzer, M.; Whiting, B. T.; Coates, G. W. Regioselective Carbonylation of trans-Disubstituted Epoxides to β-Lactones: A Viable Entry into syn-Aldol-Type Products. *J. Am. Chem. Soc.* **2013**, *135*, 10930–10933.
2. Pachfule, P.; Chen, Y.; Sahoo, S. C.; Jiang, J.; Banerjee, R. Structural Isomerism and Effect of Fluorination on Gas Adsorption in Copper-Tetrazolate Based Metal Organic Frameworks. *Chem. Mater.* **2011**, *23*, 2908–2916.
3. Sur, A.; Jernigan, N. B.; Powers, D. C. Kinetic Probes of the Origin of Activity in MOF-Based C–H Oxidation Catalysis. *ACS Catal.* **2022**, *12*, 3858–3867.
4. Orlandi, M.; Tosi, F.; Bonsignore, M.; Benaglia, M. Metal-Free Reduction of Aromatic and Aliphatic Nitro Compounds to Amines: A $HSiCl_3$-Mediated Reaction of Wide General Applicability. *Org. Lett.* **2015**, *17*, 3941–3943.
5. Gao, Y.; Yan, M.; Cheng, C.; Zhong, H.; Zhao, B.-H.; Liu, C.; Wu, Y.; Zhang, B. Membrane-Free Electrosynthesis of Epichlorohydrins Mediated by Bromine Radicals over Nanotips. *J. Am. Chem. Soc.* **2024**, *146*, 714–722.
6. Barrett, R. R. G.; Campbell, D. A.; Gleason, J. L. An Organocatalytic Oxy-Cope/Michael Cascade Reaction. *Org. Lett.* **2023**, *25*, 777–781.
7. Ji, L.; Wang, Y.-N.; Qian, C.; Chen, X.-Z. Nitrile-Promoted Alkene Epoxidation with Urea–Hydrogen Peroxide (UHP). *Synth. Commun.* **2013**, *43*, 2256–2264.
8. Mitchell, J. K.; Hussain, W. A.; Bansode, A. H.; O'Connor, R. M.; Wise, D. E.; Choe, M. H.; Parasram, M. Photoinduced Nitroarenes as Versatile Anaerobic Oxidants for Accessing Carbonyl and Imine Derivatives. *Org. Lett.* **2023**, *25*, 6517–6521.
9. Shan, Y.; Wang, A.; Zhang, S.; Liang, D.; He, M.; Zhou, W. An efficient and convenient reaction system based on MnAl layered double oxide for the aerobic oxidation of alkylarenes. *Tetrahedron Lett.* **2025**, *155*, 155426.
10. Paolillo, J. M.; Duke, A. D.; Gogarnoiu, E. S.; Wise, D. E.; Parasram, M. Anaerobic Hydroxylation of C($sp^3$)–H Bonds Enabled by the Synergistic Nature of Photoexcited Nitroarenes. *J. Am. Chem. Soc.* **2023**, *145*, 2794–2799.
